# Willingness to pay for National Health Insurance Services and Associated Factors in Africa and Asia: a systematic review and meta-analysis

**DOI:** 10.3389/fpubh.2024.1390937

**Published:** 2024-04-19

**Authors:** Ewunetie Mekashaw Bayked, Abebe Kibret Assfaw, Husien Nurahmed Toleha, Segenet Zewdie, Gebeyaw Biset, Demilade Olusola Ibirongbe, Mesfin Haile Kahissay

**Affiliations:** ^1^Department of Pharmacy, College of Medicine and Health Sciences (CMHS), Wollo University, Dessie, Ethiopia; ^2^Department of Psychology, Institute of Teachers’ Education and Behavioral Science, Wollo University, Dessie, Ethiopia; ^3^Department of Pharmacy, College of Medicine and Health Science, Injibara University, Injibara, Ethiopia; ^4^Department of Pediatrics and Child Health Nursing, College of Medicine and Health Sciences (CMHS), Wollo University, Dessie, Ethiopia; ^5^Department of Community Medicine, University of Medical Sciences, Ondo, Nigeria; ^6^Department of Pharmaceutics and Social Pharmacy, School of Pharmacy, College of Health Sciences, Addis Ababa University, Addis Ababa, Ethiopia

**Keywords:** Health insurance, National Health Insurance, willingness to pay, Factor, Africa, Asia

## Abstract

**Background:**

Universal health coverage (UHC) is crucial for public health, poverty eradication, and economic growth. However, 97% of low- and middle-income countries (LMICs), particularly Africa and Asia, lack it, relying on out-of-pocket (OOP) expenditure. National Health Insurance (NHI) guarantees equity and priorities aligned with medical needs, for which we aimed to determine the pooled willingness to pay (WTP) and its influencing factors from the available literature in Africa and Asia.

**Methods:**

Database searches were conducted on Scopus, HINARI, PubMed, Google Scholar, and Semantic Scholar from March 31 to April 4, 2023. The Joanna Briggs Institute’s (JBI’s) tools and the “preferred reporting items for systematic reviews and meta-analyses (PRISMA) 2020 statement” were used to evaluate bias and frame the review, respectively. The data were analyzed using Stata 17. To assess heterogeneity, we conducted sensitivity and subgroup analyses, calculated the Luis Furuya-Kanamori (LFK) index, and used a random model to determine the effect estimates (proportions and odds ratios) with a *p* value less than 0.05 and a 95% CI.

**Results:**

Nineteen studies were included in the review. The pooled WTP on the continents was 66.0% (95% CI, 54.0–77.0%) before outlier studies were not excluded, but increased to 71.0% (95% CI, 68–75%) after excluding them. The factors influencing the WTP were categorized as socio-demographic factors, income and economic issues, information level and sources, illness and illness expenditure, health service factors, factors related to financing schemes, as well as social capital and solidarity. Age has been found to be consistently and negatively related to the WTP for NHI, while income level was an almost consistent positive predictor of it.

**Conclusion:**

The WTP for NHI was moderate, while it was slightly higher in Africa than Asia and was found to be affected by various factors, with age being reported to be consistently and negatively related to it, while an increase in income level was almost a positive determinant of it.

## Introduction

### Context

Earth, formed 4.6 billion years ago, is divided into seven continents: Asia, Africa, North America, South America, Antarctica, Europe, and Australia (1). Asia is the largest and most populous continent (2). Asia’s vast geographical area offers a population advantage, comprising 4.6 billion out of the global population of 7.7 billion (3). The Asian region comprises a diverse array of countries, including some of the world’s least and most developed nations (4). The East Asia and Pacific region, with over two billion people, is the most populous globally, home to fast-growing economies and the second-largest number of fragile states after Africa (5).

Healthcare systems in Asia are diverse (6–8), with Japan, Singapore, South Korea, and Taiwan renowned for advanced systems, high care standards, and UHC (9–11). Governments in some Asian countries ensure healthcare access through public funding and provider regulation (12), while other Asian countries face challenges like limited access, inadequate infrastructure, and disparities in healthcare delivery (9). As a result, Asian countries are prioritizing healthcare infrastructure investment, quality access, and addressing non-communicable diseases (13, 14), while promoting collaboration and knowledge-sharing to improve health outcomes (15). Hence, Asia’s healthcare systems are undergoing significant transformations and reforms to improve access, quality, and affordability of services for their unique challenges (16, 17).

Africa is the world’s second-largest and second-most populous continent, after Asia. It spans approximately 30.3 million km^2^, including adjacent islands, accounting for 6.0% of Earth’s surface area and 20.0% of its land area. With a 2021 population of 1.4 billion, it comprises about 18.0% of the global human population (18). The majority of the African population, comprising 53.3%, is rural, with a median age of 18.8 years (19), the youngest population worldwide (18).

Healthcare systems in Africa encounter multiple challenges, such as institutional, human resources, financial, technical, and political issues (20). Africa is grappling with a significant number of both communicable and non-communicable diseases (21). The African continent was home to a particularly diverse and deadly set of tropical diseases (20). Cost-effective interventions to prevent disease burden are available, but their coverage is limited by weaknesses in health systems (21).

### Background and rationale

The primary objective of an efficient health system is to improve public health (22, 23), which critically requires UHC (22). The concept of UHC was introduced in 2005 with the aim of addressing disparities in access to healthcare services (24). The post-2015 sustainable development agenda proposes UHC as an umbrella health goal, aiming for universal, equitable, and effective delivery of comprehensive health services (25), which is at the heart of contemporary efforts to strengthen health systems (26).

However, financing UHC is a challenging task, requiring countries to consider all revenue sources for healthcare system reform (27). On the other hand, while increased spending can improve health outcomes, improving the efficiency of these expenditures is even more critical (28). Health financing involves the collection of revenue, pooling of risk, and purchasing of goods and services to enhance population health, primarily funded by individuals and households through the tax system (29). Not only revenue collection but also effective healthcare purchasing and proper regulation of healthcare providers are crucial for the sustainability of healthcare financing (30). Health purchasing is the transfer of funds to health providers, either passively or strategically, to deliver services (31).

As mentioned before, one of the targets of the Sustainable Development Goals (SDGs) is the promise to work toward achieving UHC by 2030 (32). UHC not only contributes to achieving SDG 3, good health and wellbeing, but also poverty eradication, work and economic growth, and reduced inequalities, which represent the targets of SDGs 1, 8, and 10, respectively (33). It should shield households from financial risk, especially the poorest who find it difficult to pay for services (34), which include access to high-quality, vital medical care as well as safe, effective, and reasonably priced medicines and vaccinations (32). The World Health Organization (WHO) emphasizes the significance of equitable access to safe and affordable medicines for optimal health, aligning with the SDGs, particularly SDG 3.8, which aims for UHC, and SDG 3.b, which focuses on the accessibility of medicines to address existing treatment gaps (35).

Yet, 2 billion people worldwide have no access to essential medicines (35) and face catastrophic or impoverished health spending (36), and at least 400 million individuals do not have access to essential health services (32), because the most common method of paying for health services globally is OOP spending (37). On average, each nation spends roughly 32.0% of its total health budget on OOP expenses. Due to OOP medical expenses, 150 million individuals each year experience financial catastrophe (32), and 100 million are forced into poverty (32, 38). These individuals reside in developing countries where the health systems are plagued by inefficiency, unequal access, insufficient funding, and substandard services and account for 92.0, 68.0, and 80.0% of the world’s annual deaths from communicable diseases, non-communicable conditions, and injuries, respectively (39).

**Figure 1:**
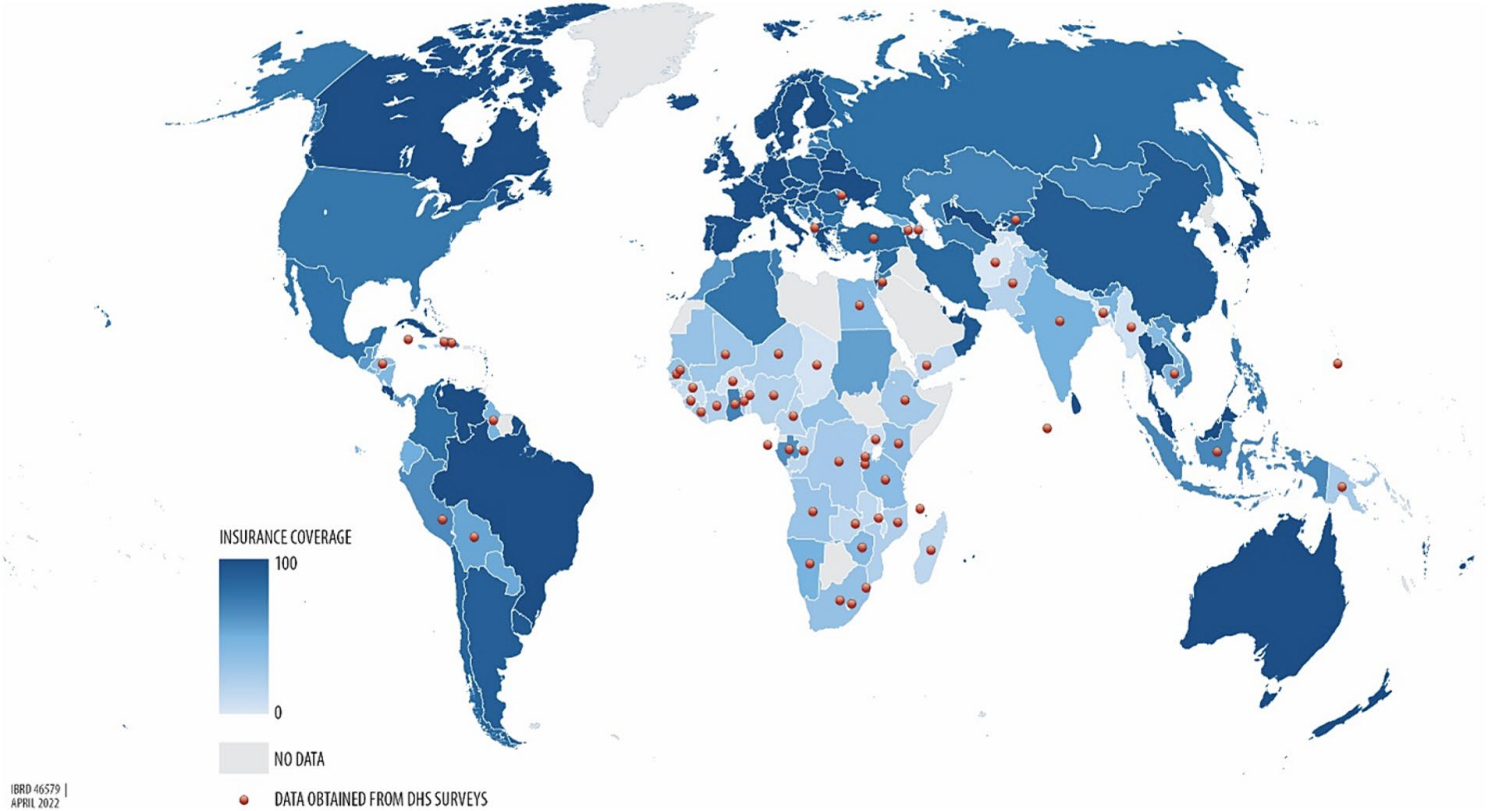
Percentage of health insurance coverage across the world, from the lowest UHC (light blue) to the highest UHC (dark blue) (40).

The burden of the lack of UHC is highest in LMICs, particularly in Africa and Asia (Figure 1) (40), where 97.0% of the population is impoverished by OOP health spending (33). In 2023, 75.0% of the 3.1 billion people worldwide without effective UHC are from LMICs in South Asia, Southeast Asia, East Asia, and Sub-Saharan Africa (SSA) (41). For example, by using their household income to access healthcare services, medications, and other products, an estimated 11 million Africans fall into poverty each year (33), which is a concerning issue considering that Asia and Africa together constitute over 75% of the global population (41).

Nevertheless, a system that generates net savings by eliminating profit and waste can be used to address the rapidly rising health expenditures (42), because up to one fifth of health expenditures could be directed towards better use by avoiding waste (43). To ensure equitable access through such a mechanism, health care must be funded, managed, and provided in a way that puts the needs of individuals and communities first (32), which is embedded in national, regional, and international contexts (44). This implies that improving health system performance to attain UHC requires actions at national, regional, and global levels (45). In fact, the dedication to collaborating with the global health community enhances access to quality healthcare and makes UHC a reality for patients, families, and communities worldwide. Together, this effort will lead to healthier communities and stronger economies (46).

Accordingly, to overcome the challenge to achieve UHC, the two regions, Africa and Asia, have recognized the importance of collaboration among governments, civil society, and the private sector. Consequently, they have started working together to use a global south perspective. Kenya and Egypt, for instance, are looking for guidance on free basic healthcare through health insurance from Thailand and Japan, respectively (33), which is, undoubtedly, the most significant sort of insurance (47).

Insurance is a contract where the subscriber pays the insurer on a regular basis in exchange for the assurance of indemnification against specific risks. The specific risks covered by health insurance are the financial burden of the treatments required following an illness or injury (48). There are several reasons to introduce health insurance, including the removal of financial barriers to healthcare access, providing financial protection against high medical costs, and negotiating better-quality healthcare with providers (49), which dictate that the way of funding health services is a crucial aspect of UHC (50). As a result, health policymakers must prioritize selecting the appropriate financing mechanisms for health services to achieve broader health policy goals, as this decision impacts both providers and consumers, particularly in low-income countries (LICs) where service usage rates across income groups are a significant issue (51).

Though there are various healthcare financing mechanisms to choose from, national, social, private, and community-based health insurance programs are the four main categories of health insurance programs (52, 53), dictating that health insurance programs can be privately or publicly run, cover various population subgroups, and provide different premium costs and benefit packages. The two primary types of government health insurance programs are NHI and Social Health Insurance (SHI), which are based on the Beveridge and Bismarck healthcare systems, respectively. Health insurance programs in many nations include components from both of these models, and the design varies across countries. However, in the majority of government health insurance schemes, enrollment, contributions, and payments are managed by a fully or partially independent government body (52).

Public health insurance models provide benefits through either a national health insurance fund, a national social security fund, or branches of the central government (54). NHI, as a public health insurance scheme, is thus provided by the federal government through general taxation (55), usually with mandatory coverage for all citizens (56). It is a single-payer scheme that covers all citizens and residents, with eligibility based on citizenship and residency status (57), indicating that it is the best option to ensure equity, fairness, and priorities aligned with medical needs. This strategy improves public health by providing universal access to desirable care with treatment options tailored to the patient’s needs (42).

Therefore, as health care is a human right and requires system-wide changes in financing to achieve UHC (58), a public, single-payer system is the best, most efficient, and most equitable health-care system. This is because, through partnerships with provider organizations and the use of taxes for everyone, single-payer systems enable people to serve as their own insurers. The best care to satisfy needs is then selected by the consumer, with minimal or no OOP expenses. That is, patients are partners in their care, obtaining diagnosis, treatment, and prevention services without financial restrictions (42). Hence, it is important to bear in mind that a nation’s financial resources primarily originate from its population, with the exception of external aid and natural resources (56).

Thus, NHI is funded through income-based premiums (59). The premium is the cost of insurance coverage, which is typically paid monthly or yearly (52). This cost of a health insurance plan is a key factor in its viability, which is determined by the members’ WTP (60), i.e., a stated preference that involves assigning a monetary value to the benefits of health-related goods or services (61). However, LICs face constraints in raising revenue to finance health and health insurance, as government tax revenues are only 15% of gross domestic product (GDP), compared to over 20% in higher-income countries (HICs) (56), and the tax structure is often regressive (62).

The WTP is the utmost amount of money that an individual is WTP for a service or product (52). It is a proxy measure of cost–benefit trade-offs in health insurance (63) and is one of two popular approaches for estimating the monetary value of health benefits, the other being the Human Capital (HC) approach (64). WTP is a widely-used concept in the health sector to guide policy decisions (65). The assessment of WTP can be conducted through evaluating historical healthcare utilization and expenditure data or by employing a contingent valuation (CV) approach (66), which is a survey methodology to assess the benefit or worth of a program to individuals (67). When employing the CV method to determine WTP, two general elements should be included: a hypothetical scenario and a bidding vehicle. The goal of the hypothetical scenario is to give the respondents a detailed explanation of the good or service they are being asked to pay for. Bids can be obtained in a number of ways, including open-ended or closed-ended questions, a bidding game, or a payment card (64).

Estimating the WTP is the best way to assess the expected income of health insurance schemes. This estimate is required to ensure that the cost of benefit packages does not surpass available resources to minimize the risk of bankruptcy. WTP data are, therefore, crucial for informing the design of tailored benefit packages for consumers, particularly groups or communities (68). Cross-country studies are decisive in assessing such data and the impacts of common elements of reforms adopted by many countries, as they provide a comprehensive understanding of which reforms have been successful or not (22). Thus, this systematic review and meta-analysis aimed to determine the pooled WTP for NHI and its influencing factors from the available literature in Africa and Asia.

## Methods

### Registration and protocol

The protocol for this review was registered on PROSPERO, accessible at https://www.crd.york.ac.uk/prospero/display_record.php?ID=CRD42023411411. We used the PRISMA 2020 Statement as a frame for the review (69) (Supplementary file S1). However, to pictorially present the screening process of the studies, because of its ease and clarity, we used the PRISMA 2009 flow diagram (70), while we sufficiently discussed the screening process in words in line with the PRISMA 2020 flow diagram.

### Eligibility criteria

All original and published cross-sectional studies that report the prevalence of WTP for NHI and/or factors influencing it were deemed eligible for the systematic review. We considered all English-language studies conducted in both community and institutional settings on WTP for NHI in Africa and Asia. The selection of studies was also based on several parameters including outcome variables, study population, year of the study, regional context, sample size, and response rate.

### Information sources and search strategy

Database searches were conducted on Scopus, HINARI, PubMed, Google Scholar, and Semantic Scholar from March 31 to April 4, 2023 (Supplementary file S2). Manual searches were performed on PubMed and HINARI. Conversely, Scopus, Google Scholar, and Semantic Scholar were searched using the “Publish or Perish” database searching tool, version 8 (71).

### Selection process

After excluding duplicate studies using Zotero reference manager version 6, two reviewers, EMB and HNT, independently screened the remaining studies. The selection process was meticulously conducted by these researchers. Initially, articles were refined based on their title and abstract; subsequently, a full-text revision was performed independently and then collaboratively until a consensus was reached. In cases of disagreement, a third reviewer was consulted for resolution.

### Data collection process and data items

A Microsoft Excel 2019 spreadsheet was utilized for data extraction. The outcome variables - population (study units), year of study, context, sample size, response rate, and proportions - were extracted using this spreadsheet. Two independent reviewers, EMB and HNT, extracted the data, compared their findings and reached a consensus. In cases where agreement could not be reached, a third reviewer was invited to help achieve consensus. Additionally, we reached out to the authors of the study to gather any missing information. The primary outcome of this systematic review and meta-analysis was the WTP for NHI. An additional outcome was the factors influencing the WTP for NHI.

### Study risk of bias assessment

Two reviewers, EMB and HNT, independently assessed the risk of bias in the included studies using JBI tools. The assessment focused on several criteria: inclusion in the sample, descriptions of study subjects and settings, validity and reliability of measurements, confounding factors and strategies to address them, and appropriateness of the outcome measure. The JBI’s tool with eight criteria was used to evaluate the risk of bias within the articles. Scores of 7 or higher were classified as low risk, 5–6 as medium risk, and 4 or lower as high risk. Studies identified as low and medium risk were included in the review. All inconsistencies were addressed through discussion and, if necessary, the involvement of a third reviewer.

### Effect measures and synthesis methods

For the qualitative synthesis, thematic strategies were utilized to categorically conceptualize the outcome variables. Preliminary effect measures were then calculated for the quantitative synthesis based on the qualitative synthesis using a Microsoft Excel 2019 spreadsheet. STATA 17 was employed to determine the effect estimates (proportions and odds ratios—ORs) of the WTP for NHI. Sub-group analyses were subsequently conducted to compare these effect estimates across studies focusing on the outcome variable. The overall level of statistical significance was set at a *p*-value less than 0.05 with a 95% confidence interval (CI).

### Reporting bias and certainty assessment

The study authors were contacted to obtain missing or incomplete data. Studies with incomplete data were excluded from the analysis. Heterogeneity between studies was assessed using the I^2^ statistic. The influence of each study on the overall meta-analysis was measured using percentages of weights, and subgroup analysis comparing the WTP for NHI in Asia and Africa was conducted. A sensitivity analysis was also conducted to determine the outlier studies.

Moreover, Doi plots were used to examine potential inter-study bias, also known as publication bias, which helps to calculate asymmetry using the LFK index (72). The Doi plot, which is a folded normal quantile (z-score) versus effect plot, replaces the conventional scatter (funnel) plot of precision versus effect. It provides a quick overview of study symmetry and heterogeneity when combined with the Galbraith plot (73). The visual examination involves observing the dots representing specific studies and their arrangement (74). In addition, the plots comprise the LFK index and *p*-value of Egger’s test (73). The LFK index is used to identify and measure the asymmetry of study effects (75). The closer the LFK index value is to zero, the greater the symmetry in the Doi plot. Values of the LFK index that fall outside the range of −1 to +1 suggest asymmetry, indicative of publication bias (76).

## Results

### Study selection

**Figure 2:**
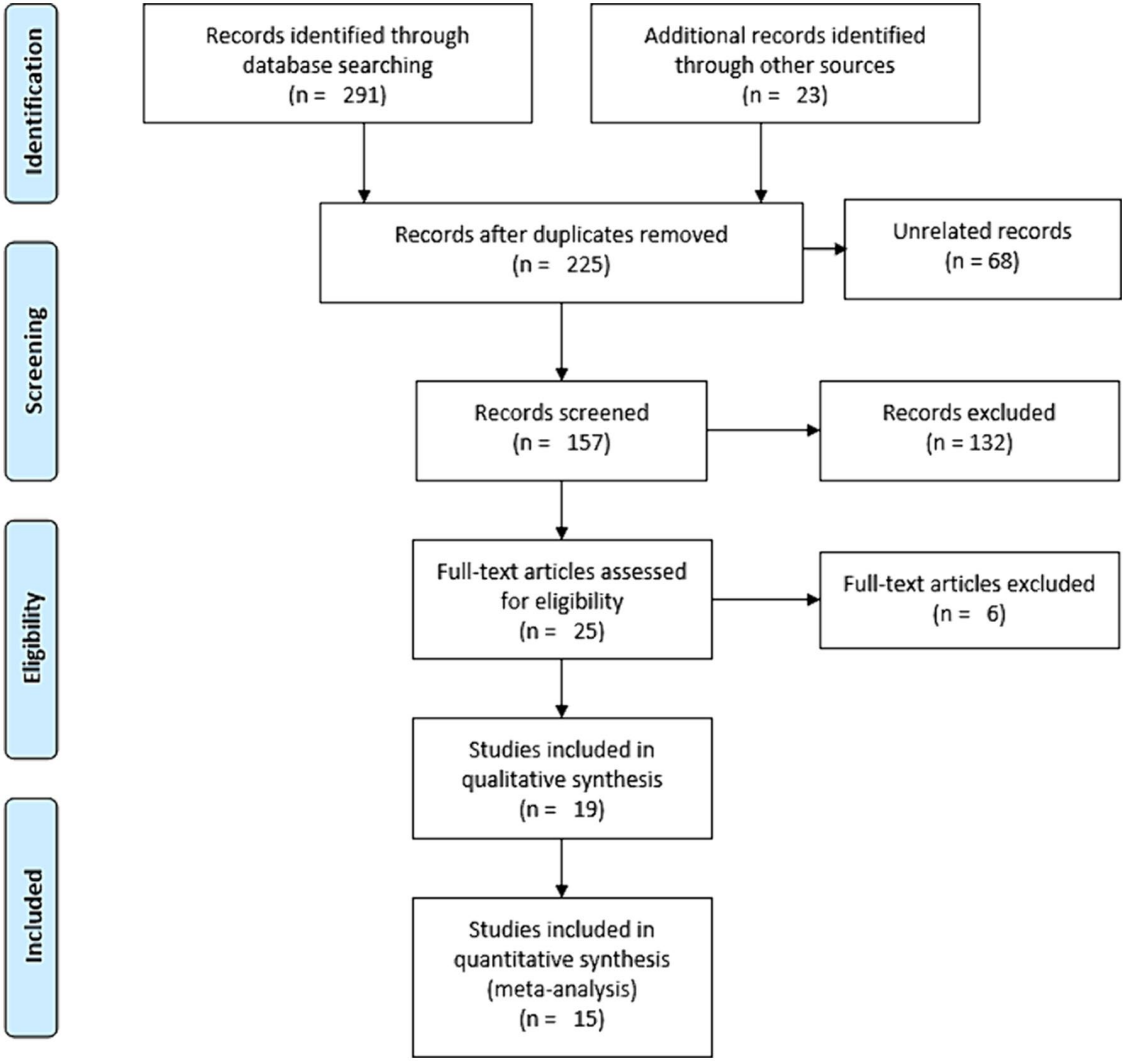
Schematic description of the screening processes for literature, 2023.

We conducted a comprehensive search of Google Scholar (*n* = 200), HINARI (*n* = 50), Scopus (*n* = 19), and PubMed (*n* = 22) from March 31 to April 4, 2023. An additional 23 records were identified through other sources, resulting in a total of 314 resources (Figure 2). After eliminating duplicates, we were left with 225 articles. Out of these, we screened 157 papers for relevance and discarded 68. Subsequently, 25 studies were chosen based on title and abstract evaluation. After a full-text evaluation, six studies were excluded due to reasons such as language other than English (77), high risk of bias (78–81), and inaccessibility of the full text (82). In conclusion, 19 resources were identified for inclusion, out of which 15 were deemed suitable for the quantitative meta-analysis.

### Study characteristics

Ten of the studies included in the systematic review were conducted in Asia, and nine were conducted in Africa. The Asian studies took place in Indonesia (*n* = 5), Malaysia (*n* = 3), and Saudi Arabia (*n* = 2). The African studies were carried out in Nigeria (*n* = 4), Gambia, South Sudan, Sierra Leone, Côte d’Ivoire, and Ghana. Each study was evaluated based on its design, context, year of study, sample size, non-response and response rates, and primary outcome. The individual characteristics of each study are summarized in Table 1.

**Table 1:** Characteristics of the included studies (*n* = 19), 2023.

Study ID	Design	Country	Continent	Year	SS	RR	Outcome
Nurlia (2021) (83)	Cross-sectional	Indonesia	Asia	2020	200	200	WTP
Oyekale (2012) (84)	Cross-sectional	Nigeria	Africa	2007	212	208	WTP
Omonira and Oyekale (2012) (85)	Cross-sectional	Nigeria	Africa	2006	122	120	WTP
Noor et al. (2019) (86)	Cross-sectional	Malaysia	Asia	2014	915	774	WTP
Oktora and Pujiyanto (2018) (87)	Cross-sectional	Indonesia	Asia	-	166	158	WTP
Omotowo et al. (2016) (88)	Cross-sectional	Nigeria	Africa	-	400	400	WTP
Al-Hanawi et al. (2018) (89)	Cross-sectional	Saudi Arabia	Asia	2015	1,250	1,187	WTP
Alharbi (2022) (90)	Cross-sectional	Saudi Arabia	Asia	2021	475	475	WTP
Njie et al. (2023) (91)	Cross-sectional	Gambia	Africa	2020	780	717	WTP
Basaza et al. (2017) (92)	Cross-sectional	South Sudan	Africa	2015	422	381	WTP
Hasan and Rahman (2022) (93)	Cross-sectional	Malaysia	Asia	2020	1,388	1,208	WTP
Agyei-Baffour et al. (2022) (94)	Cross-sectional	Sierra Leone	Africa	-	1,185	1,185	WTP
Tan (2022) (95)	Cross-sectional	Malaysia	Asia	2019	489	462	WTP
Ramadhan et al. (2015) (96)	Cross-sectional	Indonesia	Asia	2013	210	210	WTP
Oga et al. (2019) (97)	Cross-sectional	Côte d’Ivoire	Africa	2017	450	450	WTP
Oyekale and Adeyeye (2012) (98)	Cross-sectional	Nigeria	Africa	2008	110	110	WTP
Puurbalanta et al. (2020) (99)	Cross-sectional	Ghana	Africa	-	335	335	WTP
Dartanto et al. (2016) (100)	Cross-sectional	Indonesia	Asia	-	400	400	WTP
Nugraheni et al. (2022) (101)	Cross-sectional	Indonesia	Asia	2021	1,211	1,203	WTP
Total					10,720	10,183	WTP

RR, response rate; SS, sample size.

### Risk of bias in the included studies

**Figure 3:**
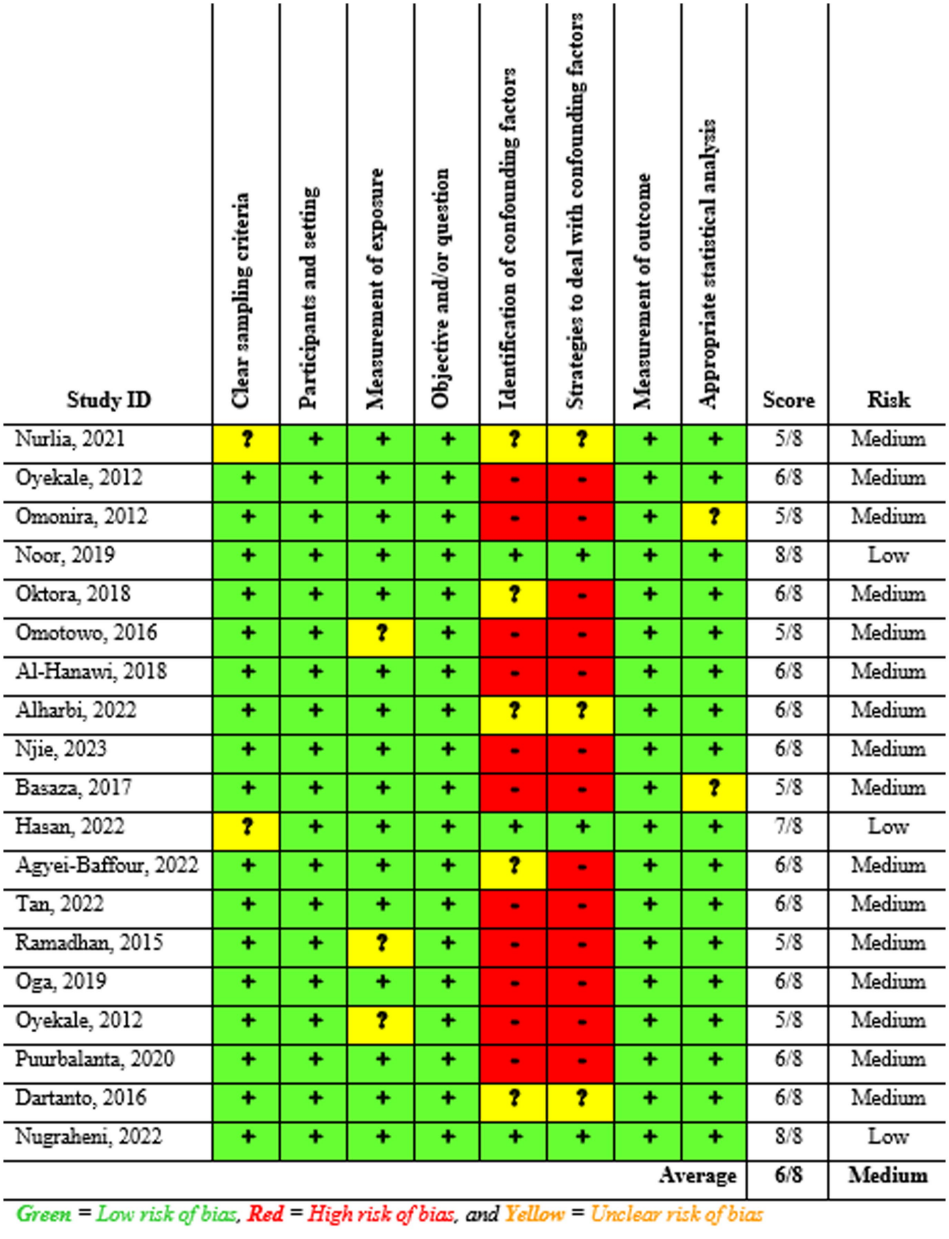
Summary of the risk of bias assessment of the included studies (*n* = 19), 2023.

The risk of bias in the included studies was evaluated using JBI’s critical appraisal tool. Subsequently, studies with low and medium risk were incorporated into the review. As depicted in (Figure 3), the average risk of bias across the studies was 6.0 (75%).

### Results of synthesis

#### Qualitative synthesis

As shown in Table 1, the sample population for all included studies (n = 19) comprised 10,720 individuals, with a response rate of 95.0%, equating to 10,183 respondents. The WTP for NHI was found to be significantly affected by various factors, which were categorized into seven themes as follows:

1. Socio-demographic factors: family size (83–85, 89, 92, 93, 100, 101), age (84, 86, 87, 90, 94, 95, 99), marital status (85, 86, 95), gender (85, 86, 88–91, 94, 95, 97, 100, 101), area of residence (86, 89, 90, 94), and education level (83, 89–91, 94, 95, 98, 99, 101).
2. Income and economic issues: level of income (83, 85–87, 89, 91, 92, 94, 99–101), extent of government taxation (85), and employment status (88, 95, 99, 101).
3. Information level and information sources: awareness about the scheme (84, 92, 94, 98), knowledge regarding the scheme (87, 95), perceptions about financing healthcare (89, 95), insurance literacy, and access to the internet (100).
4. Illness and illness expenditure: illness condition and illness experiences (84, 86, 89, 93, 95, 98, 101), and previous healthcare expenditure (100).
5. Health service factors: availability of hospitals (100), type or ownership of usual healthcare provider (90), level of health facility to get treatment, in-patient or outpatient service (101), and quality of healthcare services (89, 90).
6. Factors related to financing schemes: scheme trust and preference (84), impression of paying much more (85), having alternative health insurance (86, 89, 92, 95), and class or level of health insurance plan (101).
7. Social capital and solidarity: religious affiliation (92), level of empowerment, group and network connection, and social cohesion and inclusion (93).

As demonstrated in Table 2, the included studies reported varying results concerning the relationship between the associated variables and the WTP for NHI. Despite these inconsistencies, some studies have found consistent outcomes. Specifically, the relationship of household age and marital status with WTP for the scheme has shown a negative relationship, meaning that the WTP decreases as age and married household heads increase.

**Table 2:** Direction of the relationship between the associated factors and the WTP for NHI.

Themes (variables)	Direction of relationship with the WTP for NHI		
	Positive (+)	Negative (−)	Inconclusive
*Socio-demographic factors*			
Family size	(83, 85, 92, 101)	(84, 89, 93)	(100)
Household head’s age	–	(84, 86, 87, 90, 94, 95, 99)	–
Married participants	–	(85, 86, 95)	–
Male gender	(88, 89, 91, 97, 100)	(85, 86, 90, 94, 95, 101)	–
Place of residence (urban)	(89)	(86, 94)	(90)
Education level	(83, 89, 94, 95, 98)	–	(90, 91, 99, 101)
*Income and economic issues*			
Income level	(86, 87, 89, 94, 99–101)	(83)	(85, 91, 92)
Government taxation	–	(85)	–
Formal employment	(88, 95, 99)	(101)	–
*Information level and information sources*			
Awareness about NHI	(84, 92, 94)	(98)	–
Knowledge regarding NHI	(87)	(95)	–
Perception of financing healthcare	(95)	(89)	–
Insurance literacy	(100)	–	–
Internet access	(100)	–	–
*Illness and illness expenditure*			
Illness experience	(86, 93, 98).	(84, 89, 95, 101)	–
Previous healthcare expenditure	(100)	–	–
*Health service factors*			
Hospital availability at district level	(100)	–	–
Healthcare provider type (public hospital)	(101)	(90)	–
Service utilization (inpatient)	(101)	–	–
Health service quality	(89, 90)	–	–
*Factors related to financing schemes*			
Scheme trust and preference	(84)	–	–
Impression of paying more	–	(85)	–
Level of health insurance plan (class 1)	(101)	–	–
Having alternative health insurance	(95)	(86, 89, 92)	–
*Social capital and solidarity*			
Religious affiliation	–	–	(92)
Level of empowerment	(93)	-	–
Group and network connection	–	(93)	–
Social capital cohesion and inclusion	–	(93)	–

#### Quantitative synthesis

##### Proportional estimation of the willingness to pay

From all the included studies, 15 records with a total of 9,497 participants were included in the quantitative synthesis (Table 3). Six of the eight included studies for the meta-analysis from Asia were conducted in Indonesia (*n* = 3) and Malaysia (*n* = 3), and the rest were conducted in Saudi Arabia (*n* = 2), while those from Africa were conducted in Nigeria (*n* = 2), Gambia, Sierra Leone, Côte d’Ivoire, and Ghana. Most of the participants (62.3%) were from Asia, and the rest (37.7%) were from Africa.

**Table 3:** The frequency of the WTP for NHI in Africa and Asia (*n* = 15), 2023.

Study ID	Participants	Events (WTP)	Country	Continent
Al-Hanawi et al. (2018) (89)	1,187	826	Saudi Arabia	Asia
Alharbi (2022) (90)	475	299	Saudi Arabia	Asia
Hasan and Rahman (2022) (93)	1,208	401	Malaysia	Asia
Tan et al. (2022) (95)	462	344	Malaysia	Asia
Noor et al. (2019) (86)	774	593	Malaysia	Asia
Ramadhan et al. (2015) (96)	210	37	Indonesia	Asia
Dartanto et al. (2016) (100)	400	280	Indonesia	Asia
Nugraheni et al. (2022) (101)	1,203	496	Indonesia	Asia
Omotowo et al. (2016) (88)	400	356	Nigeria	Africa
Njie et al. (2023) (91)	717	677	Gambia	Africa
Basaza et al. (2017) (92)	381	258	South Sudan	Africa
Agyei-Baffour et al. (2022) (94)	1,185	581	Sierra Leone	Africa
Oga et al. (2019) (97)	450	350	Côte d’Ivoire	Africa
Oyekale et al. (2012) (98)	110	79	Nigeria	Africa
Puurbalanta et al. (2020) (99)	335	295	Ghana	Africa
Total	9,497	5,872	All	Both

**Figure 4:**
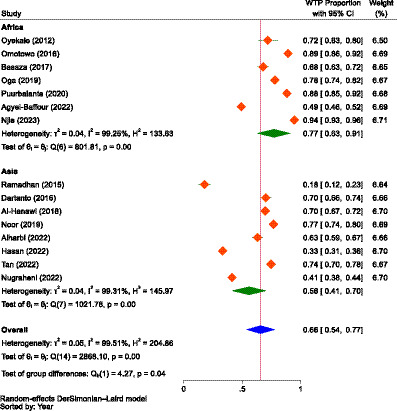
The forest plot showing the proportions of the WTP for NHI in Africa and Asia prior to excluding outliers (*n* = 15), 2023.

The combined (average) WTP for NHI in Africa and Asia was determined to be 66.0% (95% CI: 54.0–77.0%). The highest WTP for NHI was reported in Gambia at 94.0% (95% CI: 92–96%), while the lowest was reported in Indonesia at 18.0% (95% CI: 13–23%). The overall heterogeneity between studies was considerably high (102), with an I^2^ value of 99.51%. So, a random-effects model was used to calculate the pooled WTP for NHI (103). To identify the source of heterogeneity, as shown in Figure 4, we conducted a subgroup analysis based on continent, which showed significant difference between the subgroups (*p* = 0.039). According to the subgroup analysis, the WTP for NHI in Africa and Asia, respectively, was 77.0% (95% CI: 63–91%) and 56.0% (95% CI, 41–70%). The studies conducted in Sierra Leone and Gambia reported the lowest and highest WTP for the scheme in Africa, at 49.0% (95% CI, 46.0–52.0%) and 94.0% (95% CI, 92.0–96.0%), respectively. In Asia, the lowest and highest WTP for NHI were found in Indonesia and Malaysia, with rates of 18.0% (95% CI, 13–23%) and 77.0% (95% CI, 74.0–79.0%), respectively.

**Figure 5:**
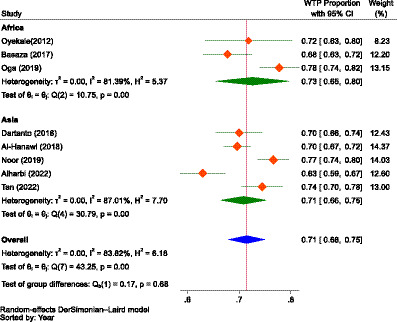
The forest plot showing the proportions of the subgroup analysis of the WTP for NHI in Africa and Asia after excluding outliers (*n* = 8), 2023.

When the outliers were excluded, the difference between the subgroups was not found to be significant (*p* = 0.680), but still, the overall result showed that there was considerable heterogeneity between studies, with an I^2^ value of 83.82%, which was significant (*p* < 0.01). After removing the outliers, the WTP for NHI in Africa decreased from 77.0% (95% CI: 63–91%) to 73.0% (95% CI: 65.0–80.0%). Conversely, the WTP for the scheme in Asia increased from 56.0% (95% CI: 41–70%) to 71.0% (95% CI: 66–75%). The combined WTP for the scheme also rose from 66.0% (95% CI: 54.0–77.0%) to 71.0% (95% CI: 68–75%; Figure 5). Consequently, the difference in the WTP levels for the scheme between the two continents narrowed. Yet, Africa’s WTP was higher than that of Asia.

##### Factors influencing the willingness to pay

Eight of the included studies reported binary outcomes regarding the influence of gender, previous illness, residence, alternative health insurance, awareness of the scheme, and the type of ownership of health facilities on the WTP for NHI. With respect to gender, two studies were from Africa and four were from Asia. About previous illness (five studies), type of ownership of facilities (two studies), and owning alternative health insurance (three studies), all studies were from Asia. Three studies, two from Asia and the other from Africa, reported about residence. Similarly, two studies, one in Africa and the other from Asia, reported awareness of the WTP for NHI. Since there was considerable heterogeneity (102) between studies and groups, we employed the DerSimonian and Laird (DL) method, which is recognized as the simplest and most widely used approach for applying the random effects model in metanalysis (104).

**Figure 6:**
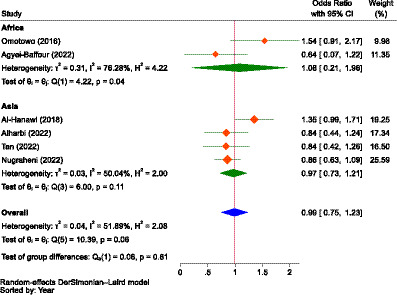
The strength of the relationship between gender and the WTP for NHI in Africa and Asia.

As demonstrated in Figure 6, the pooled estimate showed no difference between males and females concerning the WTP for NHI (OR = 0.99, 95% CI: 0.75–1.23) in Africa (Nigeria and Sierra Leone) and Asia (Saudi Arabia, Indonesia, and Malaysia). Male participants in Africa were 1.08 times more likely to pay than female individuals (OR = 1.08, 95% CI: 0.21–1.96), while in Asia, male participants were 3.0% less likely to pay for the scheme compared to female participants (OR = 0.97, 95% CI: 0.73–1.21), indicating that gender had no significant influence on WTP for NHI.

**Figure 7:**
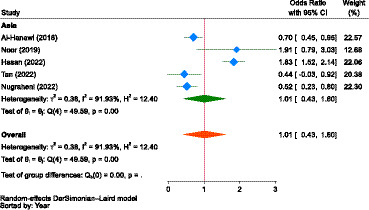
The strength of the relationship between experiencing illness and the WTP for NHI in Asia.

Regarding illness, as in the case of gender, the combined result showed that there was no significant difference between households with illness and those without (OR = 1.01, 95% CI: 0.43–1.60) in terms of likelihood to pay for NHI in Asia (Saudi Arabia, Indonesia, and Malaysia). This indicated that having a previous illness did not significantly influence the WTP for NHI (Figure 7).

**Figure 8:**
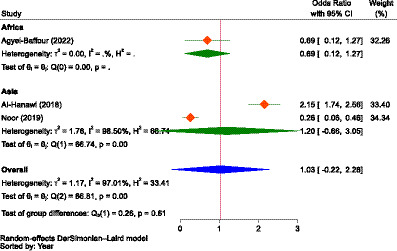
The strength of the relationship between residence and the WTP for NHI in Africa and Asia.

Concerning place of residence, the pooled estimate showed that participants living in urban areas were 1.03 times (OR = 1.03, 95% CI: 0.09–1.98) more likely to pay for the NHI scheme than those living in rural areas in Africa (Sierra Leone) and Asia (Saudi Arabia and Malaysia); however, the relationship was not significant (Figure 8). Participants living in urban areas of Africa (Sierra Leone) were 31.0% less likely to pay for the scheme compared to their rural counterparts (OR = 0.69, 95% CI: 0.62–0.76). In Asia (Saudi Arabia and Malaysia), urban residents were 1.20 times more likely to pay for the scheme (OR = 1.20, 95% CI: −0.65-3.06) than those living in rural areas.

**Figure 9:**
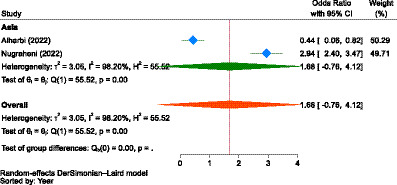
The strength of the relationship between the type of health facilities to access health services and the WTP for NHI in Asia.

The other important factor was the type of ownership of a health facility in accessing healthcare. As portrayed in Figure 9, the estimate in Asia (Saudi Arabia and Indonesia) showed that those who used healthcare services at public health facilities were 1.68 times more likely (OR = 1.68, 95% CI: −0.76-4.12) to pay for NHI compared to participants who did not use services at public health facilities, indicating that the type of ownership of the health facility had no significant influence on the WTP for NHI.

**Figure 10:**
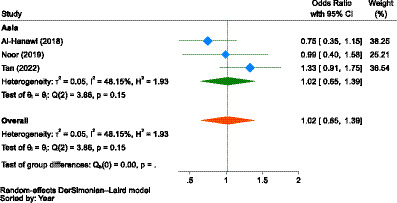
The strength of the relationship between alternative health insurance ownership and the WTP for NHI in Asia.

In Asia (Saudi Arabia and Malaysia), there was no significant difference between participants who owned PHI and those who did not (OR = 1.02, 95% CI: 0.65–1.39) to pay for NHI. However, the association between ownership of having alternative health insurance and WTP for NHI was not significant (Figure 10).

**Figure 11:**
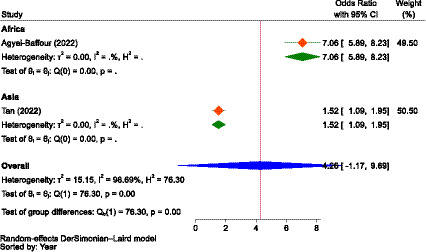
The strength of the relationship between the awareness level of participants about the scheme and the WTP for NHI in Africa and Asia.

Though the strength of association was not significant, the combined result of two studies from Africa (Sierra Leone) and Asia (Malaysia) showed that participants with good awareness about the scheme were 4.26 times more likely to pay for it (OR = 4.26, 95% CI: −1.17–9.69) than those with poor awareness. However, in both individual studies or sub-groups, awareness had a significant relationship with the WTP for NHI (Figure 11). In Asia, individuals with good awareness were 1.52 more likely (OR = 0.66, 95% CI: 0.49–0.83) to pay for NHI compared to those with poor awareness. Similarly, in Africa, those with good awareness were 7.06 times more likely (OR = 7.06, 95% CI: 5.89–8.23) to pay for it than their counterparts with poor awareness.

### Reporting bias and certainty of evidence

**Figure 12:**
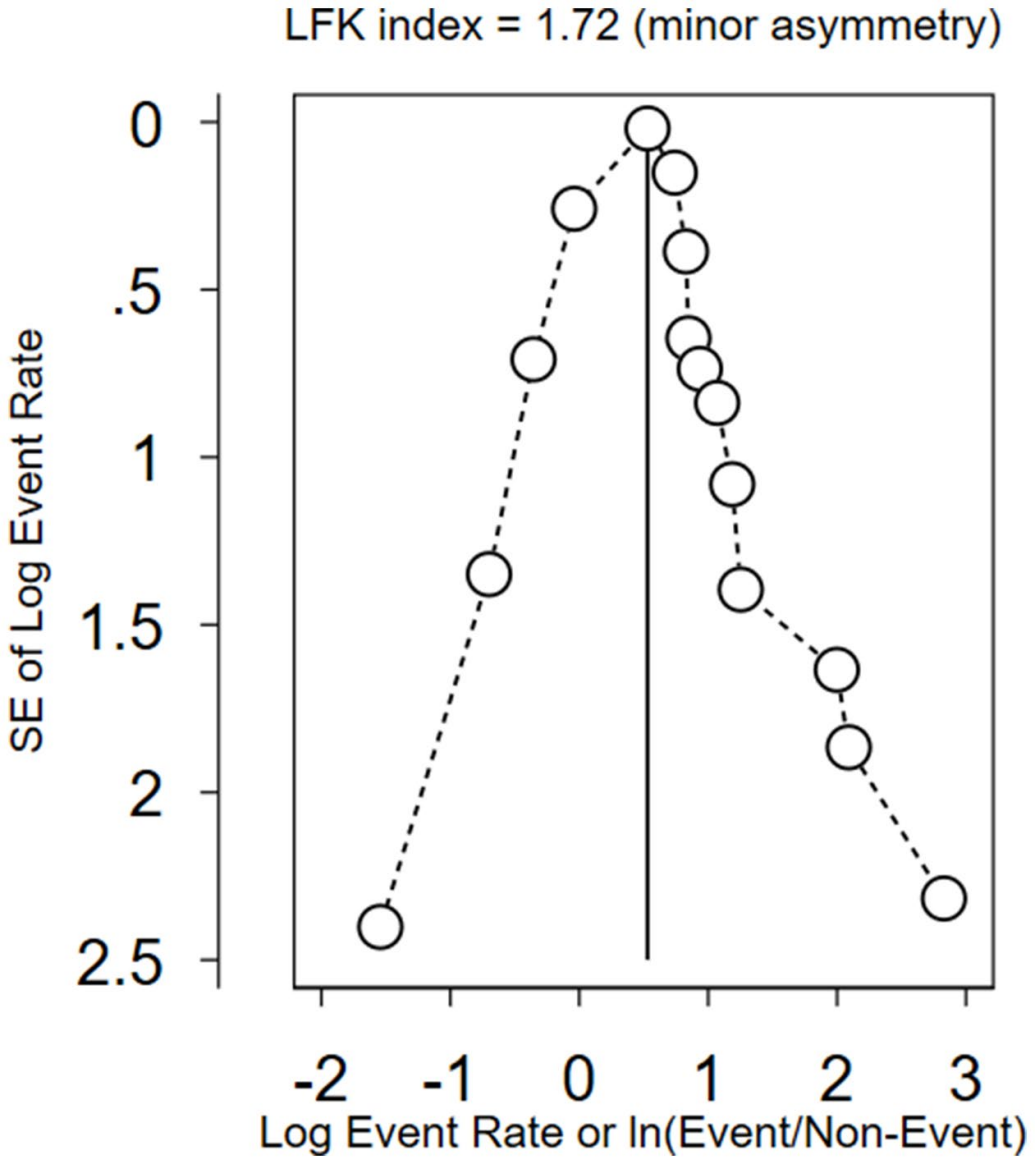
A Doi plot to assess publication bias between the included studies.

The I^2^ statistic was used to measure between-study heterogeneity, which showed that the overall I^2^ value was greater than 50% (99.51%). To control the influence of each study on the combined result, measured as percentages of weights, a random-effects model was used to calculate the pooled WTP for NHI. To identify the source of heterogeneity, we conducted a test for the subgroup difference based on continents, which was significant (*p* = 0.039). To determine if there was publication bias between studies (asymmetry or the effect of small studies), we drew a Doi plot (Figure 12) that provided an LFK index of −1.67 and showed minor asymmetry.

**Figure 13:**
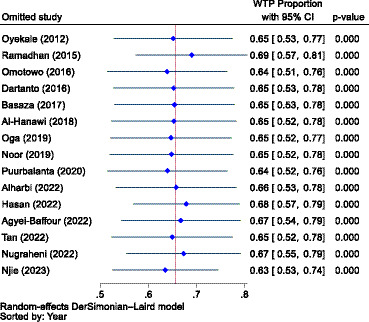
A sensitivity analysis to identify outlier studies from the included studies using the random effects model.

A sensitivity analysis was also conducted to identify outlier studies. Accordingly, seven studies were found to be outliers (88, 91, 93, 94, 96, 99, 101), and the pooled estimate (effect size) was influenced by the inclusion and exclusion of these studies. Nevertheless, as shown in Figure 13, when we adjusted for outliers using a random effects model, all of the included studies (n = 12) were found not to be outliers. Therefore, we combined the data using a random effects model without excluding any outliers. Yet, the WTP for NHI was higher in Africa compared to Asia.

## Discussion

Due to the diverse preferences of individuals, the WTP might be a very subjective measure of their intention to use health insurance. As a result, it might be affected by a variety of broad issues, like the nature of the healthcare market, information asymmetry, the psychosocial inclinations of individuals, the contexts in individual nations, the culture of a specific or defined community, health service valuation techniques or perceptions of peoples, the development level of countries, political dynamics and health policy, disease distribution and frequency, the quality of healthcare services, health system structure and taxation, reimbursement mechanisms, and the type of benefit packages to be covered, among others. The differences in the health systems of individual countries are perhaps the most crucial factors, as they shape the overall healthcare environment and are thus central to our well-being and livelihood. Should they collapse or experience disruption, it would impact the entire healthcare market and the overall sustainability of our economy (105). On the other hand, health insurance is not universally available in all developing countries, and the most cost-efficient approach to promoting health is unclear, which is a central policy concern in health economics (106). Furthermore, health insurance coverage varies greatly among different countries (107). Therefore, our discussion was not merely dependent on the specific results of this systematic review and meta-analysis; rather, we tried to deeply debate, compare, contrast, and comprehend various issues and evidence while being within the scope and context of our review and its findings.

### Prevalence of the willingness to pay

This systematic review and meta-analysis found that the pooled WTP for NHI in Africa and Asia was 71.0% (95% CI: 68–75%). Though the difference was not significant (*p* = 0.680), the WTP for the scheme in Africa (73.0, 95% CI: 65.0–80.0%) was higher than that in Asia (71.0, 95% CI: 66–75%). This is comparable to the findings of a primary study conducted in St. Vincent and the Grenadines, a Caribbean country, which reported that the WTP for NHI was 72.3% (108). From this evidence, one can easily extrapolate that the mean of the three percentages (73.0% in Africa, 71.0% in Asia, and 72.0% in Latin America) was 72.0%, which was approximately equivalent to the pooled WTP data mentioned above. This, in turn, indicates that the WTP for the scheme across the three continents was noteworthy, with minimal differences. Despite this appreciable WTP for NHI, the UHC on these continents, particularly in Africa and Asia, is still the lowest, and access to essential health services remains at a worrying level (109).

Chen et al. (110) noted that attaining UHC is difficult due to various factors globally and on these continents. Primarily, LMICs lack the funds required for UHC (110). As a result, OOP payments remained the single most common healthcare financing option in LICs, which can create a health protection gap (HPG) (111), a shortfall in finances to fund health expenditures (38). HPG is the portion of uninsured losses in total losses, or it is the sum of financially stressful OOP expenditures and the estimated cost of non-treatment due to unaffordability (111). In areas with weak financial protection, HPG, or catastrophic spending, is primarily driven by OOP payments (112). For instance, in SSA, the pooled annual incidence of catastrophic health expenditure (CHE) was 16.5%, with countries in central SSA having the highest incidence while those in southern SSA had the lowest (113). Additionally, health insurance coverage is both insufficient and unbalanced (110). These two reasons dictate that development partners should align financial and technical assistance with national health priorities, establish accountability mechanisms for resource efficiency, and strengthen national health accounts to monitor allocations and expenditures, as health financing is a global responsibility that requires solidarity and collective effort (114).

However, without controlling for outliers, the combined WTP for the scheme was 66.0% (95% CI: 54.0–77.0%), 77.0% (95% CI: 63–91%) in Africa, and 56.0% (95% CI: 41–70%) in Asia, which indicated a significant difference in the WTP for the scheme between the two continents (*p* = 0.039). In both scenarios, with and without outlier studies, the review indicated that the WTP for the scheme was higher in Africa than in Asia, which might be due to the heterogeneity in health insurance preference between the two continents’ populations, as reported by a study in Australia (115). The lower WTP for NHI in Asia might be attributable to the fact that the included studies for meta-analysis were from only three countries, as opposed to six nations in Africa. This slight lower WTP for the scheme in Asia contrasted with the evidence that consumers bear a disproportionate share of healthcare costs as OOP expenses due to inadequate UHC and the middle class’s demand for high-quality care, which in turn is expected to significantly increase their demand for health insurance (116). In Africa, even higher aggregate proportions of WTP for health insurance were reported. For instance, a cross-sectional study in seven East and West African communities revealed a 78.8% WTP for health insurance, exceeding the results of our review (60).

In fact, several African countries have recently made significant progress in extending UHC (117), as highlighted by the Africa Health Strategy 2016–2030, which aimed to strengthen national health systems as a crucial step towards achieving UHC (114). However, the rate of UHC varies significantly among countries (117). Cashin et al. (118) state that SSA is increasingly adopting public contributory health insurance for UHC, with eight countries having NHI systems and seven more considering or enacting laws. Gabon, Ghana, and Rwanda have notably extended NHI to cover large population segments, demonstrating that clear goals, equity-focused designs, strong political will, and ongoing adaptation are key to facilitating UHC through NHI (118). To that end, the WHO is collaborating with the African Union and the Africa Centers for Disease Control (CDC) to establish the African Medicines Agency (AMA). This important regulatory authority aims to improve access to medicines in Africa, where many nations struggle with pharmaceutical regulation. A vital part of this effort is guaranteeing a reliable supply of safe, effective, and high-quality medicines across the continent (119).

Nonetheless, due to the inherent drawbacks of WTP studies, it cannot necessarily be said that the demand for health insurance is higher in Africa than in Asia (120). For instance, even in Asia, an original study conducted in Indonesia reported that the ability to pay (ATP) was greater than the WTP (121). Another original study conducted in the same country showed that about 57.6% of the participants were able to pay for NHI, but only 17.4% of them were willing to pay the required premium (96). This indicates that an individual’s WTP may not align with their ability to pay (ATP), despite WTP being directly related to household income or the ATP (122). This in turn might be due to the fact that an ATP approach assumes all resources are available for healthcare, while a capacity to pay (CTP) approach considers some resources for basic needs. The CTP for healthcare is defined as a household’s consumption minus a standard amount for basic needs, known as the poverty line or basic needs line (123).

Accordingly, ATP could be a more accurate predictor of enrollment, continuity, and sustainability than WTP (122), whereas, since it is the level of contribution that would prevent an individual from falling below the poverty line, CTP could be a better indicator of the WTP (set premium amount). As a result, placing all participants together in a single pool and mandating contributions based on their CTP instead of individual or average pool risk can enable cross-subsidization and, depending on the extent of pooled resources, greatly improve financial protection for all members (124). Moreover, governments should not rely solely on household premiums as a primary source of financing but should enhance their fiscal capacity through alternative means to support an equitable healthcare system (125). This is because resilient and equitable health systems with strong population coverage are crucial for preparing for health emergencies and ensuring equity, the right to health, and justice for all people (126).

The other reason that the WTP might not be as accurate as ATP and CTP is that the responses can be biased because respondents either want to please the interviewer, indicating a high WTP, or they aim to ensure the intervention is offered at a low cost, reflecting a low WTP (127); consequently, what people say they are willing to pay may not correspond to what they actually would pay (120). The former bias is termed social desirability bias (SDB), which is a pervasive measurement challenge, particularly in the social sciences and survey research (128). This bias can increase the WTP by approximately 23–29% if surveys are administered through face-to-face interviews (129). It is more common in collectivist cultures, which tend to engage more in deception and socially desirable responses (128). That is because collectivistic cultures prioritize social harmony over personal goals, promoting forbearance and enduring adversity as coping mechanisms to maintain positive social relations (130).

Inversely, this social cohesion can have invisible benefits and can serve as a strategic tool in community-based healthcare interventions. Evidence shows that collectivistic individuals are more likely to adhere to public health interventions than their less collectivistic counterparts (131), with collectivistic leadership interventions have shown positive results (132). Hence, because healthcare spending is a critical expense for most nations and their citizens to ensure they stay healthy and receive proper care (133), and collectivism is common in the traditional societies (Asia, Latin America, and Africa) than in Western Europe, North America, and Australia (134), healthcare leaders in Africa and Asia may consider collective leadership as a strategy to achieve UHC through NHI.

Though our finding showed a higher WTP for NHI in Africa, the population generally exhibits low satisfaction levels with their healthcare systems compared to other regions globally. This persists despite significant investments from international donors and African governments, as the healthcare sector continues to struggle with issues such as limited access, corruption, infrastructural deficiencies, and medication and personnel shortages (135). On the contrary, beside the health workforce, the implementation of comprehensive NHI itself requires human resources, all of which contribute to the promotion of noble goals for everyone’s well-being (136). The primary challenge caused by funding shortages in many African countries is that their health financing systems’ strategies and mechanisms are problematic. In nearly half of these nations, household OOP payments make up 40% or more of total health expenditure, representing the least equitable method of healthcare funding (137). For instance, the health insurance coverage in the SSA is limited and favors the wealthy (138). This indicated that the majority of health care funding in SSA comes from direct OOP payments, predominantly from lower-income households (139). Donor funding is another common source of finance (139). As a result, less than half of Africa’s population (48%) has access to necessary healthcare, with the quality of services being substandard. Every year, 97 million Africans, or 8.2% of the population, face CHE, and these OOP costs drive 15 million people into poverty annually (119).

In contrast to the slightly lower WTP for NHI than Africa, estimates for Asian people were the lowest globally for delaying or foregoing care due to cost (4%) (140), because Asian individuals possess the lowest rate of being uninsured (141). Though UHC is thought to be a system that offers coverage to a vast majority of the population, the exact percentage required for it to be considered universal is debated. Some experts argue that coverage must extend to 99% of citizens and residents, whereas others believe a 90% threshold is sufficient (140). On the other hand, progress varies by country. Indonesia has advanced in health service coverage and financing, yet faces implementation challenges. Ghana’s health funding has decreased, with under 50% achievement in the UHC service coverage index. India still contends with high OOP expenses and low public health financing. Kenya still faces challenges in using public financing to enhance coverage for the informal sector, while South Africa has made little progress in strategic purchasing (142).

The disparity in population coverage could stem from the fact that many African countries adopt CBHI, characterized by fragmented coverage, whereas Asian countries prioritize SHI, which is typically more integrated (143). This demonstrates that the fragmentation and inequity caused by targeting specific population groups with various prepayment mechanisms can be addressed by harmonizing these schemes early on. Thus, it is advisable to implement strong public accountability and participation when formulating the UHC strategy (144) through equitable NHI schemes (139). In fact, most countries now prefer mixed NHI systems, learning from past experiences. These systems are not primarily funded by contributions but rather by significant tax-financed subsidies, resulting in a blend of contribution payments and government revenues. NHI schemes are thought to improve equity and reduce barriers to access in LMICs through two features: pooling, whereby financial risk is spread across the population, and pre-payment, the collection of financial resources in anticipation of service use rather than out-of-pocket payments when health care is consumed. Accordingly, a growing number of LMICs are considering rolling out NHI schemes (145).

However, there are various challenges with the implementation of NHI schemes in LMICs. Friebel et al. (145) identified several obstacles to implementing SHI or NHI programs in LMICs. Firstly, LMICs often have limited fiscal capacity compared to wealthier nations. Secondly, countries with smaller workforces may struggle to generate adequate funds for UHC. Lastly, the administrative expenses of NHI plans and the costs associated with collecting contributions are frequently overlooked (145). According to a systematic review by Lim et al. (146), the health financing challenges in Southeast Asian countries for UHC are unsustainable revenue-raising methods, fragmented health insurance schemes, a mismatch between insurance benefits and the needs of the population, political and legislative apathy, unmanageable and swiftly escalating healthcare costs, and unethical behaviors (146). A study by Oleribe et al. (147) reported that inadequate human resources, insufficient budgetary allocation to health, and poor leadership and management are key challenges facing healthcare systems in Africa on the path to UHC through health insurance (147). According to a qualitative study in Nepal, the major bottlenecks for the implementation of the NHI program included difficulty enrolling insurees, non-competitive selection of health providers, and failure to act as a prudent purchaser, leading to high dropout rates and low coverage of poor households, potentially jeopardizing the program’s sustainability if these problems persist (148).

### Factors influencing the willingness to pay

The factors affecting the WTP for NHI were thematically identified as demographic variables, income and economic issues, information level and information sources, illness and illness expenditure, health service factors, factors related to financing schemes, and social network and social solidarity. According to systematic reviews conducted in the context of LMICs on the uptake of CBHI, these factors not only affect the WTP for health insurance but also its uptake (149, 150). Another piece of evidence also showed that the choice of health insurance is influenced by a variety of factors, which can be broadly categorized into personal, economic, and external factors. Personal factors include age, health status, and income, as well as awareness, financial security, lifestyle, and risk cover. Economic factors encompass income and the cost of insurance packages, while external factors involve awareness, company-related information, risk, promotion, tax benefits, and advertising. Additionally, personality traits and clients’ preferences play a role in their choice of health insurance, often driven by social and behavioral factors (151). A systematic review of the WTP for SHI in Ethiopia also found that sociodemographic factors, health status, health service-related factors, awareness, perception, and scheme-related factors were significant determinants of the WTP (152).

#### Socio-demographic factors

Under this theme, the WTP for NHI was found to be influenced by family size, age, marital status, gender, area of residence, and level of education. Accordingly, some studies have reported that WTP for the scheme increased with family size (83, 85, 92, 101), while others have found a negative relationship between household size and WTP for the scheme (84, 89, 93). Other reviews and original studies conducted worldwide have also shown inconsistent findings regarding the relationship between family size and the WTP for NHI. For instance, a systematic review of WTP for health insurance in LMICs showed that an increase in family size was consistently correlated with higher WTP (125), while another systematic review on the uptake of health insurance in Zambia found that families with more children were less likely to contribute sustainably to health services (153). The inverse relationship between family size and the WTP for NHI may be attributed to the fact that larger families could reduce the likelihood of affording premiums (84). Or, in some countries, an extra contribution is required for each increase in family size, which may dissuade household heads from paying more (92).

This systematic review found that age was a consistent negative predictor of WTP for NHI (84, 86, 87, 90, 94, 95, 99); that is, WTP for the NHI scheme consistently decreased with increasing age. This dictates that the slightly higher WTP for NHI in Africa may be due to the continent’s youngest population, with 70% of SSA under 30 years old (154). Similarly, original studies in Germany (155), St. Vincent and the Grenadines (108), Malaysia (156), Ghana (157), and Vietnam (158), as well as systematic reviews in LMICs (125, 159), showed that the WTP for health insurance and membership was positively associated with younger age groups. However, some other original studies, such as the one conducted in Indonesia among non-salaried participants (160), Ghana (161), and in LMICs (Europe and the Eastern Mediterranean, Latin America and the Caribbean, Southeast Asia and the Western Pacific, and SSA) (162), found that older people were more likely to have health insurance and a lower probability of dropping out compared to younger adults (163). The findings of this review contradict the common belief that seniors, due to chronic illnesses or pre-existing conditions, face higher health risks and thus require increased utilization of health insurance plans (164), resulting in higher premiums than those paid by younger individuals, leading to adverse selection (165, 166). The possible reason could be that OOP payments for healthcare, which are common among older adults, significantly affect disposable income (167), which could be attributed to the inability of health insurance to provide equitable access due to limited service benefits and restricted use of services within schemes (168). Another reason may be that as people age, their supplemental income sources decrease, rendering them unable to afford the premium (169). Consequently, older individuals may have a reduced capacity to pay for necessary healthcare (170), which results in their exclusion from quality health services (171), indicating that inequity may arise in the provision of healthcare (172). Nevertheless, proactively addressing priorities can lead to the development of strategies that promote better health and equitable, goal-directed care for older adults (173).

Regarding the influence of marital status on the WTP for NHI, married households were less likely to pay compared to their single or unmarried counterparts (85, 86, 95). A study in SSA also revealed that married women’s health insurance coverage is only 21.3%, with the highest and lowest coverage in Ghana (66.7%) and Burkina Faso (0.5%). Women with household decision-making autonomy had higher odds of health insurance enrollment compared to those without such autonomy (174). A study in China found that marriage positively impacts participation in commercial health insurance, suggesting that married residents are more likely to invest in such insurance (175).

The reports on the influence of gender on WTP for NHI from the included studies were inconsistent, akin to those concerning family size. The pooled odds ratio (OR = 0.99, 95% CI: 0.75–1.23) also showed no difference between males and females in the WTP for NHI. Some studies indicated that households headed by females had a higher likelihood of WTP for NHI (85, 86, 90, 94), whereas other studies found that males were more inclined to pay (91, 97, 100, 101), which was similar to an original study in Burkina Faso showing that men were more willing to pay to join health insurance than women (176).

The report on the included studies concerning the influence of place of residence on WTP for NHI was inconsistent, similar to that of family size and gender, as evidenced by the pooled odds ratio (OR = 1.03, 95% CI: 0.09–1.98). Some of the included studies showed that the WTP for the scheme was negatively related to urban residence (86, 94), while some studies showed the reverse (89), and the other one found an inconclusive finding (90). In another study, health insurance coverage is generally higher in urban areas (177).

The other important sociodemographic variable affecting the WTP for NHI was level of education, in that in most of the included studies, participants who have higher education attainment were more likely to pay for the scheme (83, 89, 94, 95, 98), while in some of them, its influence was found to be inconclusive (90, 91, 101). Similar studies in Malaysia also showed that a higher education level was associated with a higher demand (156) and a higher WTP (125) for health insurance. Another study also found that more educated people were more likely to have health insurance (162), indicating that educational interventions can increase demand for health insurance schemes (178).

#### Income and economic issues

Factors such as income level, government taxation extent, employment status, and occupation type were categorized under this theme. Most of the included studies (86, 87, 89, 94, 99–101) showed that the WTP for NHI was higher among those with a higher income; the interest to pay for NHI was found to be increased with the household’s income level. Other studies also found that higher income was one of the most positive factors influencing the WTP for health insurance (179–181). In fact, the *per capita* level of healthcare expenditure is closely linked to the level of *per capita* income (182), because households with high incomes are more likely to be able to contribute to or pay for health insurance (183), indicating that the wealthy are more likely to be insured in most countries (177). Health insurance schemes in LMICs, despite government efforts, are often not reaching underserved populations and primarily supporting better-off groups (184). However, the health insurance system should ensure healthcare costs are proportional to households’ ability to pay, protect the poor from financial shocks, and improve service accessibility for the poor (185). Yet, governments can effectively reach marginalized and vulnerable populations in LMICs by implementing supportive regulatory, policy, and administrative provisions (186). Though trade-offs are inevitable, there are opportunities to simultaneously improve access, affordability, and equity (187).

Regarding employment status, the studies’ reports were not consistent. Some studies found that those families with a formally employed head were more likely to pay (88, 95, 99), while others reported the opposite (101). Employment status significantly influences an individual’s WTP for health insurance, as higher-income individuals are more likely to afford premiums. This can be evidenced by a study conducted in Ghana, which showed that as income increases or the number of unemployed household members decreases, people are willing to pay higher health insurance premiums (188).

#### Information level and information sources

Awareness about the scheme, knowledge regarding the scheme, and perceptions about financing healthcare, insurance literacy, and access to the internet were classified under this theme. These variables serve as motivational factors to encourage individuals to adhere to health insurance contribution payments (189). On the other hand, lack of access to information is a significant contributor to the inequality in health insurance subscriptions (190). Our review revealed that awareness level (84, 92, 94), good knowledge (87) and perception (95), insurance literacy, and access to the internet (100) were positively related to the WTP for NHI. However, some studies reported that level of awareness (98), good knowledge (95), and good perception (89) were negatively related to the WTP for the scheme. Though it was not significant, from the pooled ORs of two of the included studies, households with awareness of the scheme were 4.26 times more likely to pay for it (OR = 4.26, 95% CI: 1.17–9.69) than those without awareness. In support of our finding, a study conducted in Uganda revealed that awareness plays a crucial role in determining the demand for health insurance (191). Another study also showed that media exposure significantly contributed to the pro-rich distribution of health insurance coverage (182). However, studies showed that, for instance, in Nigeria, awareness of health insurance was low (192).

#### Illness and illness expenditure

Illness condition, illness experiences, and previous healthcare expenditure were important variables determining the WTP for NHI. In some of the included studies (84, 89, 95, 101), illness experience was negatively related to the WTP for NHI, while in others (86, 93, 98), it was positively related. The pooled OR from five of the included studies also revealed that illness experience did not significantly influence the WTP for NHI (OR = 1.01, 95% CI: 0.43–1.60). According to a study in Vietnam, decision-making regarding healthcare expenditure hinges heavily on an individual’s health status and their certainty about the future (193).

Experience of previous healthcare expenditure for healthcare services (inpatient and outpatient) was positively related to the WTP for NHI (100). This might be due to the fact that past high healthcare expenses have increased awareness of financial risks, leading to an increased WTP for health insurance and a reminder of potential medical care needs. However, a cross-sectional study among seven communities in East and West Africa showed that previous spending on healthcare was found to decrease the likelihood of being willing to pay for a health insurance scheme (60), which could be associated with poor quality health services.

#### Health service factors

These factors included the availability of hospitals, type of usual healthcare provider, place of treatment, inpatient and/or outpatient service, and quality of healthcare services. Hospital availability at district level (100), healthcare provider type (public hospital) and health service utilization (inpatient) (101), and health service quality (89, 90) were found to be positively related to the WTP for NHI. The pooled OR from two of the included studies showed that those who used healthcare services at public health facilities were 1.68 times more likely (OR = 1.68, 95% CI: −0.76-4.12) to pay for NHI compared to participants who did not use services at public health facilities, though it was not significant.

Another review also emphasized the importance of preserving health services’ equitability, affordability, and quality as crucial features (194). However, the lack of access to and unaffordable healthcare remain significant challenges faced by households worldwide (195), particularly in Africa, which faces numerous challenges in accessing medicines (196). For instance, in Egypt, governmental health expenditure accounts for one-third of total health expenditure, with OOP expenditure accounting for over 60.0% and the Ministry of Finance contributing 37.0% (197), which disproportionately burdens the poor (198). This showed that equity in financing health systems is hindered by direct payments, inadequate insurance coverage, and insufficient tax exemptions (199). Yet, NHI can improve health service accessibility and financial protection, especially for low-income individuals (200). This is because increased health insurance coverage generally leads to an increase in access to healthcare facilities (201) and can effectively manage service disparities if standardized benefit packages are implemented (202).

The UHC Coalition and WHO have incorporated quality as the fourth dimension of UHC (203), because it can significantly enhance the likelihood of achieving desired health outcomes (204). On the other hand, poor-quality services hinder UHC (205). Quality of care is therefore crucial for UHC and can be achieved through good leadership, robust planning, and intelligent investment in all settings (206). This implies that a comprehensive healthcare system should meet local needs, prioritize high-quality primary care, and involve individuals and communities in service design, delivery, assessment, and improvement (205). As a result, a well-designed national quality policy is crucial for countries to enhance health service access and outcomes, as quality is a multifaceted concept requiring strategic interventions (207). Unless, health insurance may improve structural quality but not quality measures, service delivery efficiency, or equitable benefit distribution (208).

#### Factors related to financing schemes

Factors related to financing schemes, such as trust in the scheme, insurance preference, having alternative health insurance, the impression of paying, and the type of health insurance plan, can greatly affect the WTP for NHI. Scheme trust and preference (84), level of health insurance plan (101), and having alternative health insurance (95) were found to influence the WTP for NHI positively, while the impression of paying more (85) and having alternative health insurance (86, 89, 92) were found to influence it negatively. Another study also found that enrolling in another health insurance scheme reduced the WTP for the scheme (60). However, the pooled OR from three of the included studies indicated that there was no significant difference between participants who owned alternative health insurance and those who did not (OR = 1.02, 95% CI: 0.65–1.39) to pay for NHI.

Another review also found that scheme trust, low and flexible contribution rates, benefit packages, government subsidies, and the quality of scheme administration significantly influence enrolment and contributions (183). The findings of our review differ from those of a Ugandan study, which revealed that patients without microfinance schemes are 76% less likely to enroll in a NHI scheme (209). In any way, the sub-Saharan and southeast Asian regions face challenges in health insurance development, including demand, supply, and regulatory aspects (210, 211). There are also unfavorable attitudes towards health insurance coverage even in developed countries, with 60% of Americans believing the federal government is responsible for ensuring UHC (212).

#### Social capital and solidarity

The review found that level of empowerment was found to be positively linked with the WTP for NHI, while group and network connection and social capital cohesion and inclusion were negatively linked with it (93). Another study reported that demand for health insurance is affected by social networks (213), while a study in Nigeria identified cultural and religious norms and poor social infrastructure as common barriers to adopting the NHI (214).

### Policy and practical implications

The combination of social insurance and taxes in healthcare financing systems in LMICs is known to be effective when supported by political commitment and family-based membership, contributing to rapid population coverage and leading to UHC. Effective healthcare purchasing and provider regulation are also crucial for sustainability (30), as successful universal healthcare schemes follow standard country-wide rules, combining decentralized financial management with centralized oversight and risk pooling (117). Based on the review results, it is strongly recommended to consider economic factors, particularly income levels and age stratification, in the design characteristics of the scheme and implementation strategies. Since all successful schemes offer free health coverage for the poorest segments (117), considering these factors in designing, implementing, monitoring, and evaluating is highly recommended to minimize adverse selection when determining eligibility for indigent services among poor families.

### Limitations

The data was pooled despite the high heterogeneity between the reports of the included studies. We could not be able to calculate and pool the exact amount of the WTP in monetary value due to the differences in the exchange rates between the countries. As a result, we only pooled the percentage of participants who were found to be willing to pay. Additionally, since the included studies were few in number, the result could not necessarily be generalizable to all other countries on the continents other than the countries from which the studies were included.

### Directions for future research

Since the WTP for a health intervention before any actual health adversity or experience is highly influenced by an individual’s feelings and intentions, comprehensive exploratory studies regarding the perceptions and sociocultural beliefs of NHI in Africa and Asia may be important. It might also be important to conduct actuarial analyses to measure the success of NHI implementation, which may help evaluate the effectiveness of the scheme and revise the design and implementation strategies.

## Conclusion

The WTP for NHI in Africa and Asia was moderate, while it was slightly higher in Africa than Asia. The factors affecting it were thematically identified as demographic variables, income and economic issues, information level and information sources, illness and illness expenditure, health service factors, factors related to financing schemes, and social network and social solidarity. Age was found to be consistently and negatively related to the WTP for NHI, while higher income level was almost consistently and positively related to it, which might in turn indicate that income level might decrease with increased age due to a decrease in economic productivity.

## Data availability statement

The original contributions presented in the study are included in the article/Supplementary material, further inquiries can be directed to the corresponding author.

## Author contributions

EMB: Conceptualization, Data curation, Formal analysis, Investigation, Methodology, Project administration, Resources, Software, Supervision, Validation, Visualization, Writing – original draft, Writing – review & editing. AKA: Conceptualization, Data curation, Formal analysis, Investigation, Methodology, Project administration, Resources, Software, Supervision, Validation, Visualization, Writing – original draft, Writing – review & editing. HNT: Conceptualization, Data curation, Formal analysis, Investigation, Methodology, Project administration, Resources, Software, Supervision, Validation, Visualization, Writing – original draft, Writing – review & editing. SZ: Resources, Supervision, Validation, Visualization, Writing – review & editing. GB: Resources, Supervision, Validation, Visualization, Writing – review & editing. DOI: Resources, Supervision, Validation, Visualization, Writing – review & editing. MHK: Resources, Supervision, Validation, Visualization, Writing – review & editing.

## References

[1] Devarakonda M. The World’s Largest Continent Makes Up 60% of the Global Population. Here’s Which It Is. New York, United States: Gannett Satellite Information Network (2023).

[2] Ropelato J. Top 7 Largest Continents in the World. Utah, United States: WhiteClouds (2023).

[3] Rosenberg M. Ranking the 7 Continents by Size and Population ThoughtCo (2020).

[4] Mundy L Trowman R Kearney B. Overcoming the barriers to achieving universal health Care in the Asian Region. Int J Technol Assess Health Care. New York, United States: Dotdash Meredith. (2018) 34:352–9. doi: 10.1017/S0266462318000417, PMID: 29986782

[5] Coady D. Clements B.J. Gupta S., Health financing Systems in East Asia and the Pacific: Early Successes and Current Challenges, the Economics of Public Health Care Reform in Advanced and Emerging Economies. Washington, DC: International Monetary Fund (2012). pp. 133–155.

[6] Tham TY Tran TL Prueksaritanond S Isidro JS Setia S Welluppillai V. Integrated health care systems in Asia: an urgent necessity. Clin Interv Aging. (2018) 13:2527–38. doi: 10.2147/CIA.S185048 30587945 PMC6298881

[7] Chongsuvivatwong V Phua KH Yap MT Pocock NS Hashim JH Chhem R . Health and health-care systems in Southeast Asia: diversity and transitions. Lancet. (2011) 377:429–37. doi: 10.1016/S0140-6736(10)61507-3, PMID: 21269685 PMC7159068

[8] Gauld R Ikegami N Barr MD Chiang T-L Gould D Kwon S. Advanced Asia's health systems in comparison. Health Policy. (2006) 79:325–36. doi: 10.1016/j.healthpol.2006.01.009, PMID: 16517000

[9] Wagstaff A. Health systems in East Asia: what can developing countries learn from Japan and the Asian tigers? Health Econ. (2007) 16:441–56. doi: 10.1002/hec.1180, PMID: 17066428

[10] Zhang X Oyama T. Investigating the health care delivery system in Japan and reviewing the local public hospital reform. RMHP. (2016) 9:21–32. doi: 10.2147/RMHP.S93285 PMC4807930 27051323

[11] Hong K Laude AMC Huibin A. Towards a comparative analysis of health systems reforms in the Asia-Pacific region. Asia Pacific. J Public Health. (2002) 14:9–16. doi: 10.1177/101053950201400104 12597512

[12] Yeoh E-K Johnston C Chau PYK Kiang N Tin P Tang J. Governance functions to accelerate Progress toward universal health coverage (UHC) in the Asia-Pacific region. Health Syst Reform. (2019) 5:48–58. doi: 10.1080/23288604.2018.1543521, PMID: 30924745

[13] Caussy D. U. Than sein, East Asia and Pacific states, health systems of In: Quah SR, editor. International Encyclopedia of Public Health. 2nd ed. Oxford: Academic Press (2017). 387–95.

[14] Hays R Pong LT Leopando Z. Primary care in the Asia-Pacific region: challenges and solutions. Asia Pac Fam Med. (2012) 11:8. doi: 10.1186/1447-056X-11-8, PMID: 23039696 PMC3499232

[15] Van Minh H Pocock NS Chaiyakunapruk N Chhorvann C Duc HA Hanvoravongchai P . Progress toward universal health coverage in ASEAN. Glob Health Action. (2014) 7:25856. doi: 10.3402/gha.v7.25856 25476931 PMC4256544

[16] Lin V Leung G Carter B. Asia-Pacific countries moving toward universal health coverage. Health Syst Reform. (2019) 5:1–6. doi: 10.1080/23288604.2018.1543537, PMID: 30924752

[17] Tulchinsky TH Varavikova EA Cohen MJ. Chapter 13 - national health systems In: Tulchinsky TH Varavikova EA Cohen MJ, editors. The New Public Health. 4th ed. San Diego: Academic Press (2023). 875–986.

[18] Elwakil R Ocama P Kayamba V Fouad Y Ojo O. Editorial: global excellence in gastroenterology: Africa. Front Med (Lausanne). (2022) 9:1121276. doi: 10.3389/fmed.2022.1121276 36698811 PMC9869266

[19] Worldometer. Population of Africa. Dover, United States: Worldometer (2024).

[20] Atta IA Duah HO. Better or worse? Despite Progress, many Africans still finds it difficult in accessing health care: a comparative analysis of eight African countries. J Health Med Inform. (2018) 9:299. doi: 10.4172/2157-7420.1000299

[21] Kirigia JM Barry SP. Health challenges in Africa and the way forward. Int Arch Med. (2008) 1:27. doi: 10.1186/1755-7682-1-27, PMID: 19094201 PMC2615747

[22] Gupta S. The challenge of health care reform in advanced and emerging economies In: The Economics of Public Health Care Reform in Advanced and Emerging Economies. Eds. Clements B. Coady D. Gupta S. Washington, DC: International Monetary Fund (2012). 3–21.

[23] Coady D Clements BJ Gupta S Coady D Clements BJ Gupta S. The Economics of Public Health Care Reform in Advanced and Emerging Economies Washington, DC: International Monetary Fund (2012).

[24] Wagle K. Universal Health Coverage (UHC): Dimensions, Guiding Principles and Advantages!! San Francisco, United States: Public Health Notes, WordPress (2018).

[25] Vega J Frenz P. Integrating social determinants of health in the universal health coverage monitoring framework. Rev Panam Salud Publica. (2013) 34:468–72. PMID: 24569977

[26] World Health Organization. Making Fair Choices on the Path to Universal Health Coverage: Final Report of the WHO Consultative Group on Equity and Universal Health Coverage. Geneva, Switzerland: World Health Organization (2014).

[27] Yazbeck AS Savedoff WD Hsiao WC Kutzin J Soucat A Tandon A . The case against labor-tax-financed social health insurance for Low- and Low-middle-income countries. Health Aff. (2020) 39:892–7. doi: 10.1377/hlthaff.2019.00874, PMID: 32364862

[28] Coady D. Public health care spending: past trends In: The Economics of Public Health Care Reform in Advanced and Emerging Economies. Eds. Clements B. Coady D. Gupta S. Washington, DC: International Monetary Fund (2012). 23–36.

[29] Addis Ababa University. University of Gondar, and Jimma University, Module 6: Health Care Financing, Jhpiego and Its Licensors. Milton Keynes, England: The Open University (2014).

[30] Kwon S. Thirty years of national health insurance in South Korea: lessons for achieving universal health care coverage. Health Policy Plan. (2009) 24:63–71. doi: 10.1093/heapol/czn037, PMID: 19004861

[31] Gatome-Munyua A Sieleunou I Sory O Cashin C. Why is strategic purchasing critical for universal health coverage in sub-Saharan Africa? Health Syst Reform. (2022) 8:e2051795. doi: 10.1080/23288604.2022.2051795, PMID: 35446198

[32] World Health Organization. Universal Health Coverage, World Health Organization | African Region. Geneva, Switzerland: World Health Organization (2021).

[33] Dimitrova D Akinola S. How Africa and Asia Are Joining Forces on Universal Healthcare. Davos, Switzerland: World Economic Forum, Holtzbrinck Publishing Group (2019).

[34] Beattie A Yates R Noble D. Accelerating Progress Towards Universal Health Coverage for Women and Children in South Asia. Kathmandu, Nepal: East Asia and the Pacific, UNICEF Regional Office South Asia (2016). 68 p.

[35] Chattu VK Singh B Pattanshetty S Reddy S. Access to medicines through global health diplomacy. Health Promot Perspect. (2023) 13:40–6. doi: 10.34172/hpp.2023.05, PMID: 37309432 PMC10257564

[36] World Health Organization. Universal Health Coverage (UHC). Geneva, Switzerland: World Health Organization (2023).

[37] Savedoff W., Tax-Based Financing for Health Systems: Options and Experiences, Geneva, Switzerland, World Health Organization, (2004), pp. 1–22.

[38] Swiss Re Institute. The Health Protection Gap in Asia: A modelled Exposure of USD 1.8 Trillion. Zürich, Switzerland: Swiss Re Institute (2018). 21 p.

[39] Escobar M.-L. Griffin C.C. Shaw R.P., Why and How Are We Studying Health Insurance in the Developing World? The Impact of Health Insurance in Low- and Middle-Income Countries, The Brookings Institution, Washington, DC, (2010), pp. 1–12.

[40] Das J Do Q-T. The Prices in the Crises. Washington, D.C: Centre for Economic Policy Research (CEPR) (2023).

[41] Kodali PB. Achieving universal health coverage in Low- and middle-income countries: challenges for policy post-pandemic and beyond. Risk Manag Healthc Policy. (2023) 16:607–21. doi: 10.2147/rmhp.S366759, PMID: 37050920 PMC10084872

[42] A. American Public Health. Adopting A Single-Payer Health System. Washington, DC: American Public Health Association (2021).

[43] World Health Organization. Access to Medicines and Vaccines Report by the Director-General, Seventy-Second World Health Assembly. Geneva, Switzerland: World Health Organization (2019). 8 p.

[44] Bigdeli M Jacobs B Tomson G Laing R Ghaffar A Dujardin B . Access to medicines from a health system perspective. Health Policy Plan. (2012) 28:692–704. doi: 10.1093/heapol/czs108 23174879 PMC3794462

[45] World Health Organization. Healthy Systems for Universal Health Coverage – A Joint Vision for Healthy Lives, World Health Organization and International Bank for Reconstruction and Development. Geneva, Switzerland: The World Bank (2017).

[46] Shah M. Universal Health Coverage: Collaboration to Achieve Health for All, Organisation for Economic Co-operation and Development (OECD). France: Paris Cedex (2023).

[47] Mint L. 5 Different Types of Insurance Policies & Coverage that You Need. MintLife Blog. Oakland, California: Credit Karma (2020).

[48] D'Ambrosio C Ghislandi S. Health insurance: economic and risk aspects In: Wright JD, editor. International Encyclopedia of the Social and Behavioral Sciences. 2nd ed. Oxford: Elsevier (2015). 640–5.

[49] Devadasan N Goswamy SP. Framework for Developing Health Insurance Programmes. Government of India, New Delhi, India: Ministry of Health and Family Welfare (2007).

[50] Cherry AL Baltag V Dillon ME. International Handbook on Adolescent Health and Development. Switzerland: The Public Health Response. Springer Internatioanl Publishing (2017).

[51] Jowett M. Theoretical Insights Into the Development of Health Insurance in Low-Income Countries. Centre for Health Economics: University of York (2004). 31 p.

[52] USAID. Health Insurance, Financing Roadmap, Health Policy Plus. Washington, DC: USAID (2021).

[53] Bayked EM Toleha HN Kebede SZ Workneh BD Kahissay MH. The impact of community-based health insurance on universal health coverage in Ethiopia: a systematic review and meta-analysis. Glob Health Action. (2023) 16:2189764. doi: 10.1080/16549716.2023.2189764, PMID: 36947450 PMC10035959

[54] Ly MS Bassoum O Faye A. Universal health insurance in Africa: a narrative review of the literature on institutional models. BMJ Glob Health. (2022) 7:e008219. doi: 10.1136/bmjgh-2021-008219, PMID: 35483710 PMC9052052

[55] Insuranceopedia. National Health Insurance. Edmonton, Canada: Insuranceopedia (2018).

[56] Wang H. Switlick K. Ortiz C. Zurita B. Connor C., Design Element 2. Choice of Financing Mechanisms, Africa Health Insurance Handbook—How to Make It Work, Health Systems 20/20 Project, Abt Associates Inc., Washington, DC, (2010), pp. 13–20.

[57] USAID. Health Insurance, Health Policy Plus. Washington, DC: USAID (2021).

[58] Berkhout E Oostingh H. Health Insurance in Low-Income Countries: Where Is The Evidence that It Works? Nairobi, Kenya: Oxfam International (2008). 29 p.

[59] Wen H-C Lee L-H Valvi N Dixon BE. Health information exchange in Taiwan: multiple layers to facilitate broad access and use of data for clinical and population health In: Dixon BE, editor. Health Information Exchange: Navigating and Managing a Network of Health Information Systems. Cambridge, Massachusetts: Academic Press (2023). 621–45.

[60] Bolarinwa OA Ameh S Ochimana C Oluwasanu AO Samson O Mohamed SF . Willingness and ability to pay for healthcare insurance: a cross-sectional study of seven communities in east and West Africa (SevenCEWA). PLoS Glob Public Health. (2021) 1:e0000057. doi: 10.1371/journal.pgph.0000057, PMID: 36962252 PMC10021733

[61] Abbas SM Usmani A Imran M. Willingness to pay and its role in health economics. J. Bahria Univ. Med. Dent. Coll. (2019) 9:62–6. doi: 10.51985/JBUMDC2018120

[62] Preker AS Langenbrunner J Jakab M. Rich-poor differences in health care financing, social reinsurance: a new approach to sustainable community health financing. Int. Bank Reconstr. Dev. (2002):21–36.

[63] Nzowa PG Nandonde FA Seimu SML. Mediation effect of trust on willingness to pay for health insurance among co-operative members in Tanzania. Fut Bus J. (2023) 9:18. doi: 10.1186/s43093-023-00198-0

[64] Rascati K.L., Cost-Benefit Bnalysis, Essentials of Pharmacoeconomics, Lippincott Williams & Wilkins, A Wolters Kluwer Business Philadelphia, PA, (2014), pp. 103–130.

[65] Russell S Fox-Rushby J Arhin D. Willingness and ability to pay for health care: a selection of methods and issues. Health Policy Plan. (1995) 10:94–101. doi: 10.1093/heapol/10.1.94, PMID: 10141627

[66] Brenzel L. Newbrander W., Linking ability and Willingness to Contribute to Microinsurance, Social Reinsurance: A New Approach to Sustainable Community Health Financing, Geneva, Switzerland: The International Bank for Reconstruction and Development/The World Bank and the International Labour Organisation, (2002), pp. 293–302.

[67] McGhan WF. Pharmacoeconomics In: Troy D, editor. Remington: The Science and Practice of Pharmacy. Philadelphia, United States: Lippincott Williams & Wilkins (2005). 2070–81.

[68] Dror D.M., Willingness to Pay for Health Insurance, Financing Micro Health Insurance, World Scientific, New Delhi, India, (2018), pp. 113–116.

[69] Page MJ McKenzie JE Bossuyt PM Boutron I Hoffmann TC Mulrow CD . The PRISMA 2020 statement: an updated guideline for reporting systematic reviews. BMJ. (2021) 372:n71. doi: 10.1136/bmj.n71, PMID: 33782057 PMC8005924

[70] Moher D Liberati A Tetzlaff J Altman DG. Preferred reporting items for systematic reviews and meta-analyses: the PRISMA statement. PLoS Med. (2009) 6:e1000097. doi: 10.1371/journal.pmed.1000097 19621072 PMC2707599

[71] Adams D. Publish or Perish on Microsoft Windows. St. Albans, United Kingdom: Tarma Software Research Ltd. (2016).

[72] Lu J-W Huang Y-W Chen T-L. 2.4. Data Synthesis and Statistical Analysis. California, USA: Bio-Protocol LLC (2023).

[73] Xu C Furuya-Kanamori L Doi SAR. DOIPLOT: Stata Module for Visualization of Asymmetry and Heterogeneity in Meta-Analysis. Statistical Software Components: MA, USA (2021). S459011 p.

[74] Shamim MA. Real-life implications of prevalence meta-analyses? Doi plots and prediction intervals are the answer. Lancet Microbe. (2023) 4:e490. doi: 10.1016/S2666-5247(23)00096-4, PMID: 37116520

[75] Furuya-Kanamori L Barendregt JJ Doi SAR. A new improved graphical and quantitative method for detecting bias in meta-analysis. Int J Evid Based Healthc. (2018) 16:195–203. doi: 10.1097/xeb.0000000000000141, PMID: 29621038

[76] Furuya-Kanamori L Doi SA. LFK: Stata Module to Compute LFK Index and Doi Plot for Detection of Publication Bias in Meta-Analysis. MA, USA: Statistical Software Components (2020). S458762 p.

[77] Apriani M Zulkarnaian M Idris H. Analysis of willingness to pay contributions in the membership of the National Health Insurance in regency of Banyuasin. PREPOTIF Jurnal Kesehatan Masyarakat. (2021) 5:484–95. doi: 10.31004/prepotif.v5i2.1733

[78] Edoh D Brenya A. A community-based feasibility study of National Health Insurance Scheme in Ghana. Afr J Health Sci. (2002) 9:41–50. doi: 10.4314/ajhs.v9i1.30754 17298144

[79] Mulenga K. Booysen F., Inequalities in Willingness to Pay for Zambia’s National Social Health Insurance Scheme, Research Square. (2022). doi: 10.21203/rs.3.rs-2250957/v1

[80] Lang H-C Lai M-S. Willingness to pay to sustain and expand National Health Insurance services in Taiwan. BMC Health Serv Res. (2008) 8:261. doi: 10.1186/1472-6963-8-261, PMID: 19091093 PMC2635366

[81] Aji B Masfiah S Harwanti S Ulfah N. Factors affecting willingness to pay for National Health Insurance Program among informal Workers in Indonesia. Jurnal Info Kesehatan. (2023) 21:107–15. doi: 10.31965/infokes.Vol21.Iss1.940

[82] Mansur R. Subroto A., Using Tree-Based Algorithm to Predict Informal Workers' Willingness to Pay National Health Insurance after Tele-Collection, 2022 10th International Conference on Information and Communication Technology (ICoICT), (2022), pp. 23–28.

[83] Nurlia NA Murti B Tamtomo DG. Factors correlated with willingness and compliance to pay National Health Insurance Premium in Jember regency. J Health Policy Manage. (2021) 6:35–47. doi: 10.26911/thejhpm.2021.06.01.04

[84] Oyekale AS. Factors influencing households’ willingness to pay for National Health Insurance Scheme (NHIS) in Osun state. Nigeria Stud Ethno Med. (2012) 6:167–72. doi: 10.1080/09735070.2012.11886435

[85] Omonira OF Oyekale A. Households' willingness to pay (WTP) for the National Health Insurance Scheme (NHIS): the case of Ojo Local Government Area of Lagos state. Nigeria Life Sci J. (2012) 9:3873–7.

[86] Noor AA Saperi S Aljunid SM. The Malaysian community's acceptance and willingness to pay for a National Health Financing Scheme. Public Health. (2019) 175:129–37. doi: 10.1016/j.puhe.2019.07.008, PMID: 31473369

[87] Oktora R Pujiyanto. Willingness to pay for National Health Insurance among Motorcycle Taxi Driver in Depok City, Indonesia. KnE. Life Sci. (2018) 4:190–200. doi: 10.18502/kls.v4i1.1381

[88] Omotowo IB Ezeoke UE Obi IE Uzochukwu BSC Agunwa CC Eke CB . Household perceptions, willingness to pay, benefit package preferences, health system readiness for National Health Insurance Scheme in southern Nigeria. Health. (2016) 8:1630–44. doi: 10.4236/health.2016.814159

[89] Al-Hanawi MK Vaidya K Alsharqi O Onwujekwe O. Investigating the willingness to pay for a contributory National Health Insurance Scheme in Saudi Arabia: a cross-sectional stated preference approach. Appl Health Econ Health Policy. (2018) 16:259–71. doi: 10.1007/s40258-017-0366-2, PMID: 29307076 PMC5874278

[90] Alharbi A. Willingness to pay for a National Health Insurance (NHI) in Saudi Arabia: a cross-sectional study. BMC Public Health. (2022) 22:951. doi: 10.1186/s12889-022-13353-z, PMID: 35549695 PMC9103041

[91] Njie H Wangen KR Chola L Gopinathan U Mdala I Sundby JS . Willingness to pay for a National Health Insurance Scheme in the Gambia: a contingent valuation study. Health Policy Plan. (2023) 38:61–73. doi: 10.1093/heapol/czac089, PMID: 36300926 PMC9849717

[92] Basaza R Alier PK Kirabira P Ogubi D Lako RLL. Willingness to pay for National Health Insurance Fund among public servants in Juba City, South Sudan: a contingent evaluation. Int J Equity Health. (2017) 16:158. doi: 10.1186/s12939-017-0650-7, PMID: 28854972 PMC5577679

[93] Hasan H Rahman MM. Willingness to pay for the National Health Insurance Scheme: a cross-sectional study in Sarawak, Malaysia. Bangladesh journal of. Med Sci. (2022) 21:577–89. doi: 10.3329/bjms.v21i3.59571

[94] Agyei-Baffour P Jimmy AI Twum P Larbie D Boateng KA Duah IK . Socio-demographic predictors of willingness to pay for premium of National Health Insurance: a cross-sectional survey of six districts in Sierra Leone. Int J Health Policy Manag. (2022) 11:1451–8. doi: 10.34172/ijhpm.2021.50, PMID: 34124869 PMC9808351

[95] Tan RTH Abdul Rasid SZ Wan Ismail WK Tobechan J Tan ETY Yusof AN . Willingness to pay for National Health Insurance: a contingent valuation study among patients visiting public hospitals in Melaka, Malaysia. Appl Health Econ Health Policy. (2022) 20:255–67. doi: 10.1007/s40258-021-00691-z, PMID: 34927225

[96] Ramadhan AA Rahmadi AR Djuhaeni H. Ability and willingness to pay premium in the framework of National Health Insurance System. Althea Med J. (2015) 2:635. doi: 10.15850/amj.v2n4.635

[97] Oga ASS Attia-konan AR Vehi F Kouame J Koffi K. Diabetic and cardiovascular patients’ willingness to pay for upcoming national health insurance scheme in Côte d’Ivoire. Health Econ Rev. (2019) 9:8. doi: 10.1186/s13561-019-0225-y 30848393 PMC6734508

[98] Oyekale A Adeyeye A. Rural households' awareness and willingness to pay for national health insurance scheme (NHIS) in Ilesha West Local Government Area, Osun State Nigeria: a recursive bivariate probit approach. Life Sci J. (2012) 9:2086–93.

[99] Puurbalanta R Adjei M Afosaa V. Ghana’s National Health Insurance Scheme: an ordinal Probit valuation of willingness to pay higher premiums for improved services. Am J Theor Appl Stat. (2020) 9:57. doi: 10.11648/j.ajtas.20200903.15

[100] Dartanto T Rezki JF Pramono W Siregar CH Bintara U Bintara H. Participation of informal sector Workers in Indonesia's National Health Insurance System. J Southeast Asian Econ. (2016) 33:317–42. doi: 10.1355/ae33-3c

[101] Nugraheni DA Satibi S Kristina SA Puspandari DA. Factors associated with willingness to pay for cost-sharing under universal health coverage scheme in Yogyakarta, Indonesia: a cross-sectional survey. Int J Environ Res Public Health. (2022) 19:15017. doi: 10.3390/ijerph192215017, PMID: 36429734 PMC9690347

[102] Siebert M., Heterogeneity: What Is It and Why Does It Matter? Students 4 Best Evidence, London, United Kingdom: Cochrane. (2018).

[103] Cantley N., Tutorial: How To Read a Forest Plot, Students 4 Best Evidence, London, United Kingdom: Cochrane. (2016).

[104] Jackson D Bowden J Baker R. How does the DerSimonian and Laird procedure for random effects meta-analysis compare with its more efficient but harder to compute counterparts? J Stat Plan Infer. (2010) 140:961–70. doi: 10.1016/j.jspi.2009.09.017

[105] StudySmarter. Market Failure In Healthcare. Germany: StudySmarter, StudySmarter GmbH, Munich (2023).

[106] Cutler DM Zeckhauser RJ. The anatomy of health insurance In: Culyer AJ Newhouse JP, editors. Handbook of Health Economics. Amsterdam, Netherlands: Elsevier (2000). 563–643.

[107] Odipo E. The path to universal health coverage in five African and Asian countries: examining the association between insurance status and health-care use. Lancet Glob Health. (2024) 12:e123–33. doi: 10.1016/S2214-109X(23)00510-7, PMID: 38096884 PMC10716621

[108] Adams R Chou Y-J Pu C. Willingness to participate and pay for a proposed national health insurance in St. Vincent and the grenadines: a cross-sectional contingent valuation approach. BMC Health Serv Res. (2015) 15:148. doi: 10.1186/s12913-015-0806-3, PMID: 25890181 PMC4404596

[109] Institute of Tropical Medicine. Achieving Universal Health Coverage by 2030: The Health Financing and Social Protection Challenges. Antwerp, Belgium: Institute of Tropical Medicine (2023).

[110] Chen S Cao Z Wang Z Wang C. The challenging road to universal health coverage. Lancet Glob Health. (2023) 11:e1490–1. doi: 10.1016/S2214-109X(23)00373-X, PMID: 37734784

[111] The Geneva Association. Healthcare in Emerging Markets: Exploring the Protection Gaps. The Geneva Association: Geneva, Switzerland (2019). 42 p.

[112] Thomson S. Cylus J. Evetovits T., New Numbers for Europe: Financial Hardship, Can People Afford to Pay for Health Care? New evidence on financial protection in Europe, World Health Organization, Geneva, Switzerland, (2019), pp. 29–48.

[113] Eze P Lawani LO Agu UJ Acharya Y. Catastrophic health expenditure in sub-Saharan Africa: systematic review and meta-analysis. Bull World Health Organ. (2022) 100:337–351j. doi: 10.2471/blt.21.287673, PMID: 35521041 PMC9047424

[114] Union African, Africa Health Strategy 2016–2030, African Union, Addis Ababa, Ethiopia, (2016), pp. 18–19.

[115] Buchmueller TC Fiebig DG Jones G Savage E. Preference heterogeneity and selection in private health insurance: the case of Australia. J Health Econ. (2013) 32:757–67. doi: 10.1016/j.jhealeco.2013.05.001, PMID: 23770762

[116] Gräwert AB Becker M Takabe Y Bishop A Fu S. Capturing the Health Insurance Opportunity in Asia. Boston, Massachusetts: Boston Consulting Group (2023).

[117] Schwettmann J. Towards Universal Health Coverage: The Cases of Benin. Côte d’Ivoire: Ethiopia, Kenya, Senegal and Zambia, Labour and Social Justice (2022). 30 p.

[118] Cashin C Dossou J-P. Can National Health Insurance Pave the way to universal health coverage in sub-Saharan Africa? Health Systems Reform. (2021) 7:e2006122. doi: 10.1080/23288604.2021.2006122, PMID: 34965364

[119] Cullinan K. Universal Health Coverage: Only Half of Africans Have Access to Health Care. Geneva, Switzerland: Health Policy Watch (2021).

[120] Sutton SS. Pharmacoeconomics In: McGraw-Hill's NAPLEX Review Guide. New York, United States: McGraw-Hill Education (2021). 16.

[121] Intiasari AD Aji B Masfiah S Trisnantoro L Hendrartini J. A study of ability to pay and willingness to pay of National Health Insurance Voluntary Participant in rural area. ATMPH. eds. Scott Sutton S. McGraw-Hill Companies. (2019) 22:174–82. doi: 10.36295/ASRO.2019.221124

[122] Dror D.M. Preker A.S., Introduction, Social Reinsurance: A New Approach to Sustainable Community Health Financing, The International Bank for Reconstruction and Development/The World Bank and the International Labour Organisation, Geneva, Switzerland. (2002), pp. 1–17.

[123] Thomson S. Cylus J. Evetovits T., Policy-Relevant Measurement with a Focus on Equity, Can People Afford to Pay for Health Care? New Evidence on Financial Protection in Europe, World Health Organization, Geneva, Switzerland, (2019), pp. 11–27.

[124] Gottret P. Schieber G., Collecting Revenue, Pooling Risk, and Purchasing Services Health Financing Revisited: A Practioner’s Guide, The International Bank for Reconstruction and Development/The World Bank, Washington, USA, (2006), pp. 45–72.

[125] Nosratnejad S Rashidian A Dror DM. Systematic review of willingness to pay for health Insurance in Low and Middle Income Countries. PLoS One. (2016) 11:e0157470. doi: 10.1371/journal.pone.0157470, PMID: 27362356 PMC4928775

[126] Barron GC Koonin J Akselrod S Fogstad H Karema C Ditiu L . Universal health coverage is a matter of equity, rights, and justice. Lancet Glob Health. (2023) 11:e1335–6. doi: 10.1016/S2214-109X(23)00317-0, PMID: 37429304

[127] Systems for Improved Access to Pharmaceuticals Services. Applying Principles of Pharmacoeconomics to Improve Medical Product Selection and Use in Low- and Middle-Income Countries: Trainer’s Guide. SIAPS, Arlington, USA: USAID (2017).

[128] Nurumov K Hernández-Torrano D Mhamed AAS Ospanova U. Measuring social desirability in collectivist countries: a psychometric study in a representative sample from Kazakhstan. Front Psychol. (2022) 13:822931. doi: 10.3389/fpsyg.2022.822931, PMID: 35465473 PMC9020785

[129] Leggett CG Kleckner NS Boyle KJ Duffield JW Mitchell RC. Social desirability Bias in contingent valuation surveys administered through in-person interviews. Land Econ. (2003) 79:561–75. doi: 10.2307/3147300

[130] Pharr JR Dodge Francis C Terry C Clark MC. Culture, caregiving, and health: exploring the influence of culture on family caregiver experiences. ISRN. Public Health. (2014) 2014:689826:1–8. doi: 10.1155/2014/689826

[131] Leong S Eom K Ishii K Aichberger MC Fetz K Müller TS . Individual costs and community benefits: collectivism and individuals’ compliance with public health interventions. PLoS One. (2022) 17:e0275388. doi: 10.1371/journal.pone.0275388, PMID: 36327279 PMC9632888

[132] De Brún A O’Donovan R McAuliffe E. Interventions to develop collectivistic leadership in healthcare settings: a systematic review. BMC Health Serv Res. (2019) 19:72. doi: 10.1186/s12913-019-3883-x, PMID: 30683089 PMC6347820

[133] T.I. Team. What Country Spends the Most on Healthcare? New York, USA: Investopedia (2021).

[134] Triandis HC. Collectivism: cultural concerns In: Smelser NJ Baltes PB, editors. International Encyclopedia of the Social & Behavioral Sciences. Oxford: Pergamon (2001). 2227–32.

[135] Signé L., Strategies for Effective Health Care for Africa in the Fourth Industrial Revolution: Bridging the Gap between the Promise and Delivery. Cape Town, South Africa: Africa Growth Initiative. (2021).

[136] Adyas A. The Indonesian strategy to achieve universal health coverage through National Health Insurance System: challenges in human resources. Natl Public Health J. (2021) 16:221–7. doi: 10.21109/kesmas.v16i4.5440

[137] World Health Organization. State of Health Financing in the African Region. Geneva, Switzerland: World Health Organization (2013). 25 p.

[138] Edwine B Jacob K Peter N Nirmala R. Examining the level and inequality in health insurance coverage in 36 sub-Saharan African countries. BMJ Glob Health. (2021) 6:e004712. doi: 10.1136/bmjgh-2020-004712, PMID: 33903176 PMC8076950

[139] Ifeagwu SC Yang JC Parkes-Ratanshi R Brayne C. Health financing for universal health coverage in sub-Saharan Africa: a systematic review. Glob Health Res Policy. (2021) 6:8. doi: 10.1186/s41256-021-00190-7, PMID: 33641673 PMC7916997

[140] Cronin J. Countries with Free or Universal Healthcare. Hingham, USA: International Citizens Insurance (2023).

[141] Tolbert J Drake P Damico A. Key Facts about the Uninsured Population. Washington, DC: KFF (2022).

[142] Atim C Bhushan I Blecher M Gandham R Rajan V Davén J . Health financing reforms for universal health coverage in five emerging economies. J Glob Health. (2021) 11:16005. doi: 10.7189/jogh.11.16005, PMID: 34912558 PMC8645242

[143] Spaan E Mathijssen J Tromp N McBain F ten Have A Baltussen R. The impact of health insurance in Africa and Asia: a systematic review. Bull World Health Organ. (2012) 90:685–92. doi: 10.2471/blt.12.102301, PMID: 22984313 PMC3442382

[144] Tangcharoensathien V Patcharanarumol W Panichkriangkrai W Sommanustweechai A. Policy choices for progressive realization of universal health coverage; comment on “ethical perspective: five unacceptable trade-offs on the path to universal health coverage”. Int J Health Policy Manag. (2017) 6:107–10. doi: 10.15171/ijhpm.2016.99, PMID: 28812786 PMC5287926

[145] Friebel R Josephson E Forman R Calza S. Challenges of Social Health Insurance in Low- and Lower-Middle Income Countries: Balancing Limited Budgets and Pressure to Provide Universal Health Coverage. Washington, USA: Center for Global Development (2020).

[146] Lim MY Kamaruzaman HF Wu O Geue C. Health financing challenges in southeast Asian countries for universal health coverage: a systematic review. Arch Public Health. (2023) 81:148. doi: 10.1186/s13690-023-01159-3, PMID: 37592326 PMC10433621

[147] Oleribe OO Momoh J Uzochukwu BSC Mbofana F Adebiyi A Barbera T . Identifying key challenges facing healthcare systems in Africa and potential solutions. Int J Gen Med. (2019) 12:395–403. doi: 10.2147/IJGM.S223882, PMID: 31819592 PMC6844097

[148] Gurung GB Panza A. Implementation bottlenecks of the National Health Insurance program in Nepal: paving the path towards universal health coverage: a qualitative study. Int J Health Plann Manag. (2022) 37:171–88. doi: 10.1002/hpm.3301, PMID: 34505317

[149] Adebayo EF Uthman OA Wiysonge CS Stern EA. A systematic review of factors that affect uptake of community-based health insurance in low-income and middle-income countries. BMC Health Serv Res. (2015) 15:543. doi: 10.1186/s12913-015-1179-3, PMID: 26645355 PMC4673712

[150] Bayked EM Kahissay MH Workneh BD. Factors affecting the uptake of community-based health insurance in Ethiopia: a systematic review. Int J Sci Rep. (2021) 7:459–67. doi: 10.18203/issn.2454-2156.IntJSciRep20213261

[151] PubGenius. Factors Influencing in Choosing Health Insurance? SciSpace - Question. Seattle, USA: PubGenius Inc (2023).

[152] Bayked EM Toleha HN Chekole BB Workneh BD Kahissay MH. Willingness to pay for social health insurance in Ethiopia: a systematic review and meta-analysis. Front Public Health. (2023) 11:1089019. doi: 10.3389/fpubh.2023.1089019, PMID: 37033025 PMC10073487

[153] Chamileke N. A systematic review of factors affecting uptake of health Insurance in the Informal Sector in Lusaka Province. Zambia TIJPH. (2020) 8:68–74. doi: 10.21522/TIJPH.2013.08.01.Art008

[154] Mulikita JJ. Young People’s Potential, the Key to Africa’s Sustainable Development. Washington, D.C.: United Nations (2024).

[155] Hajek A Enzenbach C Stengler K Glaesmer H Hinz A Röhr S . Determinants of willingness to pay for health Insurance in Germany—Results of the population-based health study of the Leipzig research Centre for Civilization Diseases (LIFE-adult-study). Front Public Health. (2020) 8:456. doi: 10.3389/fpubh.2020.00456, PMID: 32984246 PMC7485392

[156] Ellyana Mohamad S Abd GSR Nurcholisah F. Systematic review of factors influencing the demand for medical and health insurance in Malaysia. Int J Public Health Res. (2020) 10:1242–50.

[157] Badu E Agyei-Baffour P Ofori Acheampong I Preprah Opoku M Addai-Donkor K. Households sociodemographic profile as predictors of health insurance uptake and service utilization: a cross-sectional study in a municipality of Ghana. Advances. Public Health. (2018) 2018:1–13. doi: 10.1155/2018/7814206

[158] Lofgren C Thanh NX Chuc NTK Emmelin A Lindholm L. People's willingness to pay for health insurance in rural Vietnam. Cost Effect Resour Allocat. (2008) 6:16. doi: 10.1186/1478-7547-6-16, PMID: 18691440 PMC2527552

[159] Wang T Wang W Liang J Nuo M Wen Q Wei W . Identifying major impact factors affecting the continuance intention of mHealth: a systematic review and multi-subgroup meta-analysis. NPJ Digit Med. (2022) 5:145–13. doi: 10.1038/s41746-022-00692-9, PMID: 36109594 PMC9476418

[160] Istiqomah N Mafruhah I. Determinants of payment compliance of the national health insurance among non-salaried participants. Corporate Bus Strategy Rev. (2023) 4:54–61. doi: 10.22495/cbsrv4i4art6

[161] Sarkodie AO. Effect of the National Health Insurance Scheme on healthcare utilization and out-of-pocket payment: evidence from GLSS 7. Humanit Soc Sci Commun. (2021) 8:293. doi: 10.1057/s41599-021-00984-7

[162] Chen S Geldsetzer P Chen Q Moshabela M Jiao L Ogbuoji O . Health insurance coverage in Low- and middle-income countries remains far from the goal of universal coverage. Health Aff. (2022) 41:1142–52. doi: 10.1377/hlthaff.2021.00951 35914199

[163] Van der Wielen N Falkingham J Channon AA. Determinants of National Health Insurance enrolment in Ghana across the life course: are the results consistent between surveys? Int J Equity Health. (2018) 17:49. doi: 10.1186/s12939-018-0760-x, PMID: 29685137 PMC5913914

[164] Mao W Zhang Y Xu L Miao Z Dong D Tang S. Does health insurance impact health service utilization among older adults in urban China? A nationwide cross-sectional study. BMC Health Serv Res. (2020) 20:630. doi: 10.1186/s12913-020-05489-8, PMID: 32646423 PMC7346393

[165] Li J. Effects of Age and Income on Individual Health Insurance Premiums Undergraduate Economic Review 7 (2011) 1–20.

[166] Sengupta R Rooj D. The effect of health insurance on hospitalization: identification of adverse selection, moral hazard and the vulnerable population in the Indian healthcare market. World Dev. (2019) 122:110–29. doi: 10.1016/j.worlddev.2019.05.012

[167] Scheil-Adlung X Bonan J. Gaps in social protection for health care and long-term care in Europe: are the elderly faced with financial ruin? Int Soc Secur Rev. (2013) 66:25–48. doi: 10.1111/issr.12001

[168] Amani PJ Hurtig A-K Frumence G Kiwara AD Goicolea I San Sebastiån M. Health insurance and health system (un) responsiveness: a qualitative study with elderly in rural Tanzania. BMC Health Serv Res. (2021) 21:1140. doi: 10.1186/s12913-021-07144-2, PMID: 34686182 PMC8532322

[169] Amilaku EM Fentaye FW Mekonen AM Bayked EM. Willingness to pay for social health insurance among public civil servants: a cross-sectional study in Dessie City Administration, North-East Ethiopia. Front Public Health. (2022) 10:920502. doi: 10.3389/fpubh.2022.920502, PMID: 35928482 PMC9343680

[170] Morgan AK Adei D Agyemang-Duah W Mensah AA. An integrative review on individual determinants of enrolment in National Health Insurance Scheme among older adults in Ghana. BMC Primary Care. (2022) 23:190. doi: 10.1186/s12875-022-01797-6 35907799 PMC9338578

[171] Ruano AL Rodríguez D Rossi PG Maceira D. Understanding inequities in health and health systems in Latin America and the Caribbean: a thematic series. Int J Equity Health. (2021) 20:94. doi: 10.1186/s12939-021-01426-1, PMID: 33823879 PMC8023548

[172] Van Hees SGM O’Fallon T Hofker M Dekker M Polack S Banks LM . Leaving no one behind? Social inclusion of health insurance in low- and middle-income countries: a systematic review. Int J Equity Health. (2019) 18:134. doi: 10.1186/s12939-019-1040-0, PMID: 31462303 PMC6714392

[173] Fulmer T Reuben DB Auerbach J Fick DM Galambos C Johnson KS. Actualizing better health and health care for older adults. Health Aff. (2021) 40:219–25. doi: 10.1377/hlthaff.2020.01470 33476185

[174] Zegeye B Idriss-Wheeler D Ahinkorah BO Ameyaw EK Seidu A-A Adjei NK . Association between women’s household decision-making autonomy and health insurance enrollment in sub-Saharan Africa. BMC Public Health. (2023) 23:610. doi: 10.1186/s12889-023-15434-z, PMID: 36997885 PMC10064715

[175] Xiao W. Effects of marital status on household commercial health insurance participation behavior. J Interdisc Math. (2018) 21:397–407. doi: 10.1080/09720502.2017.1420569

[176] Dong H Kouyate B Snow R Mugisha F Sauerborn R. Gender's effect on willingness-to-pay for community-based insurance in Burkina Faso. Health Policy. (2003) 64:153–62. doi: 10.1016/s0168-8510(02)00144-6, PMID: 12694952

[177] Bachmann MO. Would national health insurance it prove equity and efficiency of health care in South Africa? Lessons from Asia and Latin America. S Afr Med J. (1994) 84:153–7. 7740352

[178] Khan JAM Ahmed S. Impact of educational intervention on willingness-to-pay for health insurance: a study of informal sector workers in urban Bangladesh. Health Econ Rev. (2013) 3:12. doi: 10.1186/2191-1991-3-12, PMID: 23628206 PMC3644264

[179] Mustikasari VD Malasari AN Rochmah TN Sugiarto K. The effect of willingness to pay on customer preferences for health insurance membership: a systematic review. Jurnal Aisyah. (2023) 8:2052. doi: 10.30604/jika.v8i3.2052

[180] Panda A Lambert P Surminski S. Insurance and Financial Services Across Developing Countries: An Empirical Study of Coverage and Demand. London, United Kingdom: Grantham Research Institute on Climate Change and the Environment (2020).

[181] Javan-Noughabi J Kavosi Z Faramarzi A Khammarnia M. Identification determinant factors on willingness to pay for health services in Iran. Health Econ Rev. (2017) 7:40. doi: 10.1186/s13561-017-0179-x, PMID: 29159659 PMC5696272

[182] Rannan-Eliya R.P., Extending Social Health Protection in the Asia Pacific Region: Progress and Challenges, International Labour Organization, New Delhi, India, (2008), pp. 9–20.

[183] Miti JJ Perkio M Metteri A Atkins S. Factors associated with willingness to pay for health insurance and pension scheme among informal economy workers in low- and middle-income countries: a systematic review. Int J Soc Econ. (2020) 48:17–37. doi: 10.1108/IJSE-03-2020-0165

[184] Osei Afriyie D Krasniq B Hooley B Tediosi F Fink G. Equity in health insurance schemes enrollment in low and middle-income countries: a systematic review and meta-analysis. Int J Equity Health. (2022) 21:21. doi: 10.1186/s12939-021-01608-x, PMID: 35151323 PMC8841076

[185] Bennett S. Gilson L., Health Financing: Designing and Implementing Pro-Poor Policies. Pretoria, South Africa: Human Sciences Research Council. (2001).

[186] Shah A Lemma S Tao C Wong J. The role of health policy and Systems in the Uptake of community-based health insurance schemes in Low- and middle-income countries: a narrative review. Health Serv Insights. (2023) 16:11786329231172675. doi: 10.1177/11786329231172675, PMID: 37153878 PMC10155025

[187] Shrank WH DeParle N-A Gottlieb S Jain SH Orszag P Powers BW . Health costs and financing: challenges and strategies for a new administration. Health Aff. (2021) 40:235–42. doi: 10.1377/hlthaff.2020.01560, PMID: 33476208

[188] Asenso-Okyere WK Osei-Akoto I Anum A Appiah EN. Willingness to pay for health insurance in a developing economy. A pilot study of the informal sector of Ghana using contingent valuation. Health Policy. (1997) 42:223–37. doi: 10.1016/S0168-8510(97)00069-9, PMID: 10176302

[189] Trisnasari O Laosee CR Janmaimool P. Assessing the determinants of compliance with contribution payments to the National Health Insurance Scheme among informal Workers in Indonesia. Int J Environ Res Public Health. (2023) 20:7130. doi: 10.3390/ijerph20237130, PMID: 38063558 PMC10705999

[190] Atakorah YB Arthur E Osei-Fosu AK Novignon J. Economic inequalities in health insurance subscription renewal: evidence from Ghana's National Health Insurance Scheme. Soc Sci Med. (2023) 341:116514. doi: 10.1016/j.socscimed.2023.116514, PMID: 38142607

[191] Mpuuga D Yawe BL Muwanga J. Determinants of demand for health Insurance in Uganda: an analysis of utilisation and willingness to pay. Tanzanian Econ Rev. (2020) 10:1–22. doi: 10.56279/ter.v10i1.53

[192] Akwaowo CD Umoh I Udo AA Motilewa OO Dan E Akpan N . Willingness to join social health insurance and community-based health insurance among rural residents in Akwa Ibom state, Nigeria. Ibom Med J. (2023) 16:207–17. doi: 10.61386/imj.v16i2.320

[193] Vuong Q-H Ho T-M Nguyen H-K Vuong T-T. Healthcare consumers’ sensitivity to costs: a reflection on behavioural economics from an emerging market. Palgrave Commun. (2018) 4:70. doi: 10.1057/s41599-018-0127-3

[194] Noor Aizuddin A Sulong S Aljunid SM. Factors influencing willingness to pay for healthcare. BMC Public Health. (2012) 12:A37. doi: 10.1186/1471-2458-12-S2-A37

[195] Nwanaji-Enwerem O Bain P Marks Z Nwanaji-Enwerem P Staton CA Olufadeji A . Patient satisfaction with the Nigerian National Health Insurance Scheme two decades since establishment: a systematic review and recommendations for improvement. Afr J Prim Health Care Fam Med. (2022) 14:e1–e10. doi: 10.4102/phcfm.v14i1.3003, PMID: 35144455 PMC8831992

[196] Adebisi YA Nwogu IB Alaran AJ Badmos AO Bamgboye AO Rufai BO . Revisiting the issue of access to medicines in Africa: challenges and recommendations. Public health. Challenges. (2022) 1:e9. doi: 10.1002/puh2.9 PMC12039675 40496656

[197] Fasseeh A ElEzbawy B Adly W ElShahawy R George M Abaza S . Healthcare financing in Egypt: a systematic literature review. J Egypt Public Health Assoc. (2022) 97:1. doi: 10.1186/s42506-021-00089-8, PMID: 34994859 PMC8741917

[198] Ahmed Y Ramadan R Sakr MF. Equity of health-care financing: a progressivity analysis for Egypt. J Hum Appl Soc Sci. (2020) 3:3–24. doi: 10.1108/JHASS-08-2019-0040

[199] Rostampour M Nosratnejad S. A systematic review of equity in healthcare financing in Low- and middle-income countries. Value Health Reg Issues. (2020) 21:133–40. doi: 10.1016/j.vhri.2019.10.001, PMID: 31786404

[200] Bodhisane S Pongpanich S. The impact of National Health Insurance upon accessibility of health services and financial protection from catastrophic health expenditure: a case study of Savannakhet province, the Lao People’s Democratic Republic. Health Res Policy Syst. (2019) 17:99. doi: 10.1186/s12961-019-0493-3, PMID: 31842882 PMC6915990

[201] Erlangga D Suhrcke M Ali S Bloor K. The impact of public health insurance on health care utilisation, financial protection and health status in low- and middle-income countries: a systematic review. PLoS One. (2019) 14:e0219731. doi: 10.1371/journal.pone.0219731, PMID: 31461458 PMC6713352

[202] Debie A Khatri RB Assefa Y. Contributions and challenges of healthcare financing towards universal health coverage in Ethiopia: a narrative evidence synthesis. BMC Health Serv Res. (2022) 22:866. doi: 10.1186/s12913-022-08151-7, PMID: 35790986 PMC9254595

[203] Barker P. Making Universal Health Coverage Whole: Adding Quality as the Fourth Dimension. Orlando, USA: Institute for Healthcare Improvement (2016).

[204] World Health Organization, OECD, and International Bank for Reconstruction and Development/the World Bank, Global State of Health Care Quality, Delivering Quality Health Services: A Global Imperative for Universal Health Coverage, Geneva, Switzerland: World Health Organization, OECD, and International Bank for Reconstruction and Development/The World Bank, (2018), pp. 27–38.

[205] World Health Organization, OECD, and International Bank for Reconstruction and Development/the World Bank, Building Quality Into the Foundations of Health Systems, Delivering Quality Health Services: A Global Imperative for Universal Health Coverage, Geneva, Switzerland: World Health Organization, OECD, and International Bank for Reconstruction and Development/The World Bank, (2018), pp. 41–56.

[206] World Health Organization, OECD, and International Bank for Reconstruction and Development/the World Bank, Background: Striving for Quality in Health Care Services, Delivering Quality Health Services: A Global Imperative for Universal Health Coverage, World Health Organization, OECD, and International Bank for Reconstruction and Development/The World Bank, (2018), pp. 15–22.

[207] World Health Organization, OECD, and International Bank for Reconstruction and Development/the World Bank, Understanding Levers to Improve Quality, Delivering Quality Health Services: A Global Imperative for Universal Health Coverage, World Health Organization, OECD, and International Bank for Reconstruction and Development/The World Bank, Geneva, Switzerland. (2018), pp. 57–71.

[208] Osei Afriyie D Masiye F Tediosi F Fink G. Purchasing for high-quality care using National Health Insurance: evidence from Zambia. Health Policy Plan. (2023) 38:681–8. doi: 10.1093/heapol/czad022, PMID: 37022137 PMC10274566

[209] Kangwagye P Bright LW Atukunda G Basaza R Bajunirwe F. Utilization of health insurance by patients with diabetes or hypertension in urban hospitals in Mbarara, Uganda. PLoS Global Public Health. (2023) 3:e0000501. doi: 10.1371/journal.pgph.0000501, PMID: 37315042 PMC10266657

[210] Kellett J Dhaliwal M. Can Insurance and Telemedicine Revolutionize Healthcare in Africa? NY, USA: United Nations Development Programme (2022).

[211] Palas JU Ashraf M Ray PK. Financing universal health coverage: a systematic review. Int Technol Manage Rev. (2017) 6:133–48. doi: 10.2991/itmr.2017.6.4.2

[212] Kiley J. 60% in US Say Health Care Coverage is Government’s Responsibility. Washington, DC: Pew Research Center (2018).

[213] Oraro T Ngube N Atohmbom GY Srivastava S Wyss K. The influence of gender and household headship on voluntary health insurance: the case of north-West Cameroon. Health Policy Plan. (2018) 33:163–70. doi: 10.1093/heapol/czx152, PMID: 29145600

[214] Ewulum K Abiodun OP Ogunniyi AO Ajani OF Yashim AN Tomori MO . Enrollees’ knowledge, satisfaction, and barriers to uptake of National Health Insurance Scheme in north-Central Nigeria. MGM J Med Sci. (2022) 9:89–96. doi: 10.4103/mgmj.mgmj_79_21

