# Supplementary material for: Willingness to pay for National Health Insurance Services and Associated Factors in Africa and Asia: a systematic review and meta-analysis
Source: Front Public Health. 2024 Apr 19;12:1390937. doi: 10.3389/fpubh.2024.1390937 (PMC11066245; doi:10.3389/fpubh.2024.1390937)
Supplement: Supplementary file 1 [file Table_1.DOCX]

**Supplementary File 2: Database search**

1. **PubMed (n = 22):**

Search: **((("willingness") AND ("pay")) AND ("national health insurance")) AND (factor)**

"willingness"[All Fields] AND "pay"[All Fields] AND "national health insurance"[All Fields] AND ("factor"[All Fields] OR "factor s"[All Fields] OR "factors"[All Fields])

**Translations**

factor: "factor"[All Fields] OR "factor's"[All Fields] OR "factors"[All Fields]

**Results:** 22

**Time:** 09:15:19

**Results**

1. Chueh, C.-H. *et al.* Cost-Effectiveness Analysis of a New Second-Line Treatment Regimen for Advanced Intrahepatic Cholangiocarcinoma: Biomarker-Driven Targeted Therapy of Pemigatinib Versus 5-FU Chemotherapy. *Pharmacoeconomics* 41, 307–319 (2023).
2. Yang, M.-C., Tan, E. C.-H. & Su, J.-J. Cost-effectiveness analysis of quadrivalent versus trivalent influenza vaccine in Taiwan: A lifetime multi-cohort model. *Hum Vaccin Immunother* 13, 81–89 (2017).
3. Lee, M.-C. *et al.* Cost-effectiveness analysis of rivaroxaban plus aspirin versus aspirin alone in secondary prevention among patients with chronic cardiovascular diseases. *Cardiovasc Drugs Ther* 35, 539–547 (2021).
4. Guan, H., Wang, C., Chen, C., Han, S. & Zhao, Z. Cost-Effectiveness of 12 First-Line Treatments for Patients With Advanced EGFR Mutated NSCLC in the United Kingdom and China. *Front Oncol* 12, 819674 (2022).
5. Kandel, M., Dunant, A., Balleyguier, C. & Bonastre, J. Cost-effectiveness of preoperative magnetic resonance imaging to optimize surgery in ductal carcinoma in situ of the breast. *Eur J Radiol* 129, 109058 (2020).
6. Kim, C. H. *et al.* Direct medical costs after surgical or nonsurgical treatment for degenerative lumbar spinal disease: A nationwide matched cohort study with a 10-year follow-up. *PLoS One* 16, e0260460 (2021).
7. Kim, A.-R., Lee, S. M. & An, S. Estimating the economic value of counselling services using the contingent valuation method. *Psychother Res* 28, 820–828 (2018).
8. Nugraheni, D. A., Satibi, S., Kristina, S. A. & Puspandari, D. A. Factors Associated with Willingness to Pay for Cost-Sharing under Universal Health Coverage Scheme in Yogyakarta, Indonesia: A Cross-Sectional Survey. *Int J Environ Res Public Health* 19, 15017 (2022).
9. Holbrook, R. *et al.* Implantable cardioverter defibrillator therapy is cost effective for primary prevention patients in Taiwan: An analysis from the Improve SCA trial. *PLoS One* 15, e0241697 (2020).
10. Al-Hanawi, M. K., Vaidya, K., Alsharqi, O. & Onwujekwe, O. Investigating the Willingness to Pay for a Contributory National Health Insurance Scheme in Saudi Arabia: A Cross-sectional Stated Preference Approach. *Appl Health Econ Health Policy* 16, 259–271 (2018).
11. Badu, E., Agyei-Baffour, P., Ofori Acheampong, I., Opoku, M. P. & Addai-Donkor, K. Perceived satisfaction with health services under National Health Insurance Scheme: Clients’ perspectives. *Int J Health Plann Manage* 34, e964–e975 (2019).
12. Pan, Y.-J., Kuo, K.-H. & Wang, S.-J. Pharmacological treatment of depression with and without headache disorders: an appraisal of cost effectiveness and cost utility of antidepressants. *J Affect Disord* 170, 255–265 (2015).
13. Cho, D. & Jo, C. Preference elicitation approach for measuring the willingness to pay for liver cancer treatment in Korea. *Clin Mol Hepatol* 21, 268–278 (2015).
14. González Block, M. A., Vargas Bustamante, A., de la Sierra, L. A. & Martínez Cardoso, A. Redressing the limitations of the Affordable Care Act for Mexican immigrants through bi-national health insurance: a willingness to pay study in Los Angeles. *J Immigr Minor Health* 16, 179–188 (2014).
15. Harris, B. *et al.* Social solidarity and civil servants’ willingness for financial cross-subsidization in South Africa: implications for health financing reform. *J Public Health Policy* 32 Suppl 1, S162-183 (2011).
16. Muttaqien, M. *et al.* Why did informal sector workers stop paying for health insurance in Indonesia? Exploring enrollees’ ability and willingness to pay. *PLoS One* 16, e0252708 (2021).
17. Adams, R., Chou, Y.-J. & Pu, C. Willingness to participate and Pay for a proposed national health insurance in St. Vincent and the grenadines: a cross-sectional contingent valuation approach. *BMC Health Serv Res* 15, 148 (2015).
18. Alharbi, A. Willingness to pay for a National Health Insurance (NHI) in Saudi Arabia: a cross-sectional study. *BMC Public Health* 22, 951 (2022).
19. Njie, H. *et al.* Willingness to pay for a National Health Insurance Scheme in The Gambia: a contingent valuation study. *Health Policy Plan* 38, 61–73 (2023).
20. Basaza, R., Alier, P. K., Kirabira, P., Ogubi, D. & Lako, R. L. L. Willingness to pay for National Health Insurance Fund among public servants in Juba City, South Sudan: a contingent evaluation. *Int J Equity Health* 16, 158 (2017).
21. Tan, R. T. H. *et al.* Willingness to Pay for National Health Insurance: A Contingent Valuation Study Among Patients Visiting Public Hospitals in Melaka, Malaysia. *Appl Health Econ Health Policy* 20, 255–267 (2022).
22. Chiwire, P., Evers, S. M., Mahomed, H. & Hiligsmann, M. Willingness to pay for primary health care at public facilities in the Western Cape Province, Cape Town, South Africa. *J Med Econ* 24, 162–172 (2021).
23. **Google scholar (n = 200)** **– using “Perish or Publish” software:**

**willingness to pay "national health insurance"**

*Publish or Perish 8.8.4275.8412 (basic report)
WinPosix (x64) edition, running on WinPosix 10.0.19045 (x64)*

**Search terms**

**Keywords:** willingness to pay "national health insurance"
**Years:** all
**Other options:** include citations; include patents

**Data retrieval**

**Data source:** Google Scholar
**Search date:** 2023-03-31 14:33:18 +00300
**Cache date:** 2023-03-31 11:31:01 +00300
**Search result:** [0] No error

***Important:*** *This data source provides only abbreviated data. Any ellipses (... marks) shown in this report originate with the data source; they are NOT caused by subsequent processing in Publish or Perish.*

**Metrics**

**Reference date:** 2023-03-31 11:31:01 +00300
**Publication years:** 1971-2023
**Citation years:** 52 (1971-2023)
**Papers:** 200
**Citations:** 5415
**Citations/year:** 104.13 (acc1=125, acc2=84, acc5=37, acc10=11, acc20=2)
**Citations/paper:** 27.08
**Authors/paper:** 3.01/3.0/3 (mean/median/mode)
**Age-weighted citation rate:** 575.94 (sqrt=24.00), 217.91/author
**Hirsch h-index:** 38 (a=3.75, m=0.73, 4153 cites=76.7% coverage)
**Egghe g-index:** 70 (g/h=1.84, 4930 cites=91.0% coverage)
**PoP hI,norm:** 21
**PoP hI,annual:** 0.40
**Fassin hA-index:** 10

**Results**

1. AS Oyekale (2012) Factors influencing households' willingness to pay for National Health Insurance Scheme (NHIS) in Osun state, Nigeria. *Studies on Ethno-Medicine*, Taylor &Francis, doi:10.1080/09735070.2012.11886435, cited by 42 (3.82 per year)
2. MK Al-Hanawi, K Vaidya, O Alsharqi, ... (2018) Investigating the willingness to pay for a contributory National Health Insurance Scheme in Saudi Arabia: a cross-sectional stated preference approach. *… health economics and …*, Springer, doi:10.1007/s40258-017-0366-2, cited by 54 (10.80 per year)
3. R Basaza, PK Alier, P Kirabira, ... (2017) Willingness to pay for National Health Insurance Fund among public servants in Juba City, South Sudan: a contingent evaluation. *… for equity in …*, equityhealthj.biomedcentral.com, doi:10.1186/s12939-017-0650-7, cited by 35 (5.83 per year)
4. HC Lang, MS Lai (2008) Willingness to pay to sustain and expand National Health Insurance services in Taiwan. *BMC health services research*, Springer, doi:10.1186/1472-6963-8-261, cited by 24 (1.60 per year)
5. WK Asenso-Okyere, I Osei-Akoto, A Anum, EN Appiah (1997) Willingness to pay for health insurance in a developing economy. A pilot study of the informal sector of Ghana using contingent valuation. *Health policy*, Elsevier, cited by 283 (10.88 per year)
6. MA González Block, A Vargas Bustamante, ... (2014) Redressing the limitations of the Affordable Care Act for Mexican immigrants through bi-national health insurance: a willingness to pay study in Los Angeles. *Journal of immigrant and …*, Springer, doi:10.1007/s10903-012-9712-5, cited by 37 (4.11 per year)
7. IB Omotowo, UE Ezeoke, IE Obi, BSC Uzochukwu, ... (2016) Household perceptions, willingness to pay, benefit package preferences, health system readiness for National Health Insurance scheme in southern Nigeria. *Health*, scirp.org, cited by 17 (2.43 per year)
8. S Nosratnejad, A Rashidian, M Mehrara, ... (2014) Willingness to pay for social health insurance in Iran. *Global journal of …*, ncbi.nlm.nih.gov, cited by 57 (6.33 per year)
9. M Muttaqien, H Setiyaningsih, V Aristianti, ... (2021) Why did informal sector workers stop paying for health insurance in Indonesia? Exploring enrollees' ability and willingness to pay. *PloS one*, journals.plos.org, cited by 12 (6.00 per year)
10. AA Ramadhan, AR Rahmadi, ... (2015) Ability and willingness to pay premium in the framework of national health insurance system. *Althea Medical …*, journal.fk.unpad.ac.id, cited by 8 (1.00 per year)
11. H Njie, KR Wangen, L Chola, ... (2023) Willingness to pay for a National Health Insurance Scheme in The Gambia: a contingent valuation study. *Health Policy and …*, academic.oup.com
12. S Nosratnejad, A Rashidian, DM Dror (2016) Systematic review of willingness to pay for health insurance in low and middle income countries. *PloS one*, journals.plos.org, cited by 86 (12.29 per year)
13. R Adams, YJ Chou, C Pu (2015) Willingness to participate and Pay for a proposed national health insurance in St. Vincent and the grenadines: a cross-sectional contingent valuation …. *BMC health …*, bmchealthservres.biomedcentral …, doi:10.1186/s12913-015-0806-3, cited by 48 (6.00 per year)
14. E Gustafsson-Wright, A Asfaw, J van der Gaag (2009) Willingness to pay for health insurance: An analysis of the potential market for new low-cost health insurance products in Namibia. *Social science &medicine*, Elsevier, cited by 145 (10.36 per year)
15. RTH Tan, SZ Abdul Rasid, WK Wan Ismail, ... (2022) Willingness to Pay for National Health Insurance: A Contingent Valuation Study Among Patients Visiting Public Hospitals in Melaka, Malaysia. *… Health Economics and …*, Springer, doi:10.1007/s40258-021-00691-z, cited by 1 (1.00 per year)
16. D Mpuuga, BL Yawe, J Muwanga (2020) Determinants of demand for health insurance in Uganda: An analysis of utilisation and willingness to pay. *Tanzanian Economic Review*, journals.udsm.ac.tz, cited by 6 (2.00 per year)
17. A Kebede, M Gebreslassie, M Yitayal (2014) Willingness to pay for community based health insurance among households in the rural community of Fogera District, North West Ethiopia. *International Journal of Economics …*, Citeseer, cited by 55 (6.11 per year)
18. BR Entele, NV Emodi (2016) Health insurance technology in Ethiopia: willingness to pay and its implication for health care financing. *Am J Public Health Res*, article.scipublichealthresearch.com, cited by 17 (2.43 per year)
19. SS Habib, S Zaidi (2021) Exploring willingness to pay for health insurance and preferences for a benefits package from the perspective of women from low-income households of …. *BMC Health Services …*, bmchealthservres.biomedcentral …, doi:10.1186/s12913-021-06403-6, cited by 10 (5.00 per year)
20. HC Lang (2010) Willingness to pay for lung cancer treatment. *Value in health*, Wiley Online Library, doi:10.1111/j.1524-4733.2010.00743.x, cited by 69 (5.31 per year)
21. S Ahmed, ME Hoque, AR Sarker, M Sultana, Z Islam, ... (2016) Willingness-to-pay for community-based health insurance among informal workers in urban Bangladesh. *PloS one*, journals.plos.org, cited by 123 (17.57 per year)
22. A Kuwawenaruwa, J Macha, J Borghi (2011) Willingness to pay for voluntary health insurance in Tanzania. *East African medical journal*, ajol.info, cited by 32 (2.67 per year)
23. JAM Khan, S Ahmed (2013) Impact of educational intervention on willingness-to-pay for health insurance: A study of informal sector workers in urban Bangladesh. *Health economics review*, healtheconomicsreview …, doi:10.1186/2191-1991-3-12, cited by 55 (5.50 per year)
24. Onwujekwe, E Okereke, C Onoka, ... (2010) Willingness to pay for community-based health insurance in Nigeria: do economic status and place of residence matter?. *Health policy and …*, academic.oup.com, cited by 174 (13.38 per year)
25. B Khatiwada, S Ghimire, N Shrestha, KB Shrestha, ... (2017) Willingness to pay for health insurance in Mangalbare Village Development Committee of Illam District. *MOJ Public …*, academia.edu, cited by 6 (1.00 per year)
26. R Mansur, A Subroto (2022) Using Tree-Based Algorithm to Predict Informal Workers' Willingness to Pay National Health Insurance after Tele-Collection. *2022 10th International Conference on …*, ieeexplore.ieee.org
27. Kaonga, F Masiye, JM Kirigia (2022) How viable is social health insurance for financing health in Zambia? Results from a national willingness to pay survey. *Social Science &Medicine*, Elsevier
28. AA Shafie, MA Hassali (2013) Willingness to pay for voluntary community-based health insurance: Findings from an exploratory study in the state of Penang, Malaysia. *Social science &medicine*, Elsevier, cited by 89 (8.90 per year)
29. Batbold, C Pu (2021) Willingness to pay for private health insurance among workers with mandatory social health insurance in Mongolia. *International Journal for Equity …*, equityhealthj.biomedcentral.com, doi:10.1186/s12939-020-01343-9, cited by 12 (6.00 per year)
30. P Agyei-Baffour, AI Jimmy, P Twum, D Larbie, ... (2022) Socio-demographic predictors of willingness to pay for premium of national health insurance: a cross-sectional survey of six districts in Sierra Leone. *International Journal of …*, ijhpm.com, cited by 1 (1.00 per year)
31. MT Gidey, GB Gebretekle, ME Hogan, ... (2019) Willingness to pay for social health insurance and its determinants among public servants in Mekelle City, Northern Ethiopia: a mixed methods study. *Cost Effectiveness and …*, Springer, doi:10.1186/s12962-019-0171-x, cited by 45 (11.25 per year)
32. AA Noor, S Saperi, SM Aljunid (2019) The Malaysian community's acceptance and willingness to pay for a National Health Financing Scheme. *Public health*, Elsevier, cited by 7 (1.75 per year)
33. A Azhar, MM Rahman, MT Arif (2018) Willingness to pay for health insurance in Sarawak, Malaysia: a contingent valuation method. *Bangladesh Journal of Medical …*, banglajol.info, cited by 20 (4.00 per year)
34. ASS Oga, AR Attia-Konan, ... (2019) Diabetic and cardiovascular patients' willingness to pay for upcoming national health insurance scheme in Côte d'Ivoire. *Health …*, healtheconomicsreview …, doi:10.1186/s13561-019-0225-y, cited by 4 (1.00 per year)
35. A Alharbi (2022) Willingness to pay for a National Health Insurance (NHI) in Saudi Arabia: a cross-sectional study. *BMC Public Health*, Springer, doi:10.1186/s12889-022-13353-z
36. H Dong, B Kouyate, J Cairns, R Sauerborn (2005) Inequality in willingness-to-pay for community-based health insurance. *Health policy*, Elsevier, cited by 76 (4.22 per year)
37. WY Chen, C Chi, YH Lin (2011) The willingness to pay for the health care under Taiwan's national health insurance system. *Applied Economics*, Taylor &Francis, doi:10.1080/00036840802600228, cited by 5 (0.42 per year)
38. B Abel-Smith (1994) Employer's willingness to pay: the case for compulsory health insurance in Tanzania. *Health Policy and Planning*, academic.oup.com, cited by 37 (1.28 per year)
39. YK Ogundeji, B Akomolafe, K Ohiri, NN Butawa (2019) Factors influencing willingness and ability to pay for social health insurance in Nigeria. *PloS one*, journals.plos.org, cited by 35 (8.75 per year)
40. CD Akwaowo, I Umoh, O Motilewa, B Akpan, ... (2021) Willingness to pay for a contributory social health insurance scheme: a survey of rural residents in Akwa Ibom state, Nigeria. *Frontiers in Public …*, frontiersin.org, doi:10.3389/fpubh.2021.654362, cited by 8 (4.00 per year)
41. R Basaza, EP Kyasiimire, PK Namyalo, ... (2019) Willingness to pay for Community Health Insurance among taxi drivers in Kampala City, Uganda: a contingent evaluation. *… and Healthcare Policy*, Taylor &Francis, doi:10.2147/RMHP.S184872, cited by 15 (3.75 per year)
42. R Babatunde, O Oyedeji, AE Omoniwa, ... (2016) Willingness-to-pay for community based health insurance by farming households: a case study of hygeia community health plan in Kwara State, Nigeria. *Trakia Journal of …*, tru.uni-sz.bg, cited by 16 (2.29 per year)
43. A Minyihun, MG Gebregziabher, YA Gelaw (2019) Willingness to pay for community-based health insurance and associated factors among rural households of Bugna District, Northeast Ethiopia. *BMC research notes*, Springer, doi:10.1186/s13104-019-4091-9, cited by 35 (8.75 per year)
44. M Apriani, M Zulkarnaian, ... (2021) ANALYSIS OF WILLINGNESS TO PAY CONTRIBUTIONS IN THE MEMBERSHIP OF THE NATIONAL HEALTH INSURANCE IN REGENCY OF BANYUASIN. *PREPOTIF …*, journal.universitaspahlawan.ac.id, cited by 1 (0.50 per year)
45. E Kalyango, RM Kananura, ... (2021) Household preferences and willingness to pay for health insurance in Kampala City: a discrete choice experiment. *Cost …*, resource-allocation.biomedcentral …, doi:10.1186/s12962-021-00274-8, cited by 8 (4.00 per year)
46. Y Lasebew, Y Mamuye, S Abdelmenan (2017) Willingness to pay for the newly proposed social health insurance among health workers at St. Paul's Hospital Millennium Medical College, Addis Ababa …. *Int J Health Econ Policy*, academia.edu, cited by 12 (2.00 per year)
47. A Mekonne, B Seifu, C Hailu, A Atomsa (2020) Willingness to pay for social health insurance and associated factors among health care providers in Addis Ababa, Ethiopia. *BioMed Research …*, hindawi.com, cited by 15 (5.00 per year)
48. AA Istamayu, A Solida, ... (2022) Determinan Kemauan Membayar (Willingness to Pay) Iuran Jaminan Kesehatan Nasional pada Peserta Mandiri di Kota Jambi Tahun 2021. *AKSELERASI: Jurnal …*, ejournal.goacademica.com, cited by 2 (2.00 per year)
49. ME Banwat, HA Agbo, Z Hassan, S Lassa, ... (2012) Community based health insurance knowledge and willingness to pay; A survey of a rural community in North Central zone of Nigeria. *Jos Journal of …*, ajol.info, cited by 27 (2.45 per year)
50. DK Sunjaya, DMD Herawati, ED Sihaloho, ... (2022) Factors Affecting Payment Compliance of the Indonesia National Health Insurance Participants. *… and Healthcare Policy*, Taylor &Francis, doi:10.2147/RMHP.S347823, cited by 1 (1.00 per year)
51. AO Lawanson, MN Ibrahim (2015) Willingness to pay for community health insurance: a study of Hygeia operations in Shonga and Afon communities in Kwara State. *African Journal of Health Economics*, ajhe.org.in, cited by 5 (0.63 per year)
52. K Chang (2010) Comorbidities, quality of life and patients' willingness to pay for a cure for type 2 diabetes in Taiwan. *Public Health*, Elsevier, cited by 53 (4.08 per year)
53. I Gupta, M Trivedi (2014) Willingness to pay for health insurance among HIV-positive patients in India. *Applied health economics and health policy*, Springer, doi:10.1007/s40258-014-0105-x, cited by 9 (1.00 per year)
54. T Dartanto, JF Rezki, W Pramono, CH Siregar, ... (2016) Participation of informal sector workers in Indonesia's national health insurance system. *Journal of Southeast …*, JSTOR, cited by 40 (5.71 per year)
55. H Hasan, MM Rahman (2022) Willingness to Pay for the National Health Insurance Scheme: A Cross-sectional study in Sarawak, Malaysia. *Bangladesh Journal of Medical Science*, banglajol.info
56. WM Oo, KT Lwin, PP Soe, SH Lwin, MO Win, TZC Bo, ... (2015) Awareness and Acceptance of Health Insurance, and Willingness to Pay: a Community based Study in Myanmar., meral.edu.mm, cited by 4 (0.50 per year)
57. A Setegn, G Andargie, G Amare, ... (2021) Willingness to pay for social health insurance among teachers at governmental schools in Gondar town, Northwest Ethiopia. *Risk Management and …*, Taylor &Francis, doi:10.2147/RMHP.S298256, cited by 7 (3.50 per year)
58. KT Han, YL Yu, W Kim, S Kang (2021) Is Korean society prepared for the financial burden of novel anticancer drugs? A survey of willingness to pay among National Health Insurance beneficiaries. *Supportive Care in Cancer*, Springer, doi:10.1007/s00520-021-06091-2
59. RA Hirth, ME Chernew, E Miller, ... (2000) Willingness to pay for a quality-adjusted life year: in search of a standard. *Medical decision …*, journals.sagepub.com, doi:10.1177/0272989X0002000310, cited by 1082 (47.04 per year)
60. A Hidayat, A Razak, S Balqis, ... (2022) Determinants Of Ability And Willingness To Pay National Health Insurance Contributions To Traders At Losari Beach. *Journal of Positive …*, journalppw.com
61. Onwujekwe, EV Velenyi (2012) Willingness to pay for private voluntary health insurance in southeast Nigeria. *African journal of health economics*, ajhe.org.in, cited by 5 (0.45 per year)
62. W Tadele, M Aklilu, Y Getaneh, A Defar, Y Acham, ... (2020) Willingness to pay forsocial health insurance among staffs in Ethiopian Public Health Institute, Addis Ababa. *Ethiopian Journal of public …*, cited by 3 (1.00 per year)
63. MM Rahman, S Mizan, R binti Safii, ... (2020) Willingness to pay for health insurance among urban poor: An evidence from urban primary health care project in Bangladesh. *Bangladesh Journal of …*, banglajol.info, cited by 4 (1.33 per year)
64. S Nosratnejad, A Rashidian, AA Sari, ... (2017) Willingness to pay for complementary health care insurance in Iran. *Iranian journal of public …*, ncbi.nlm.nih.gov, cited by 11 (1.83 per year)
65. D Dakoye, RO Anguyo, P Govule, SP Katongole, ... (2015) Communities' willingness to pay for healthcare in public health facilities of Nakasongola district, Uganda., ir.umu.ac.ug, cited by 7 (0.88 per year)
66. IE Anderson, FO Adeniji (2019) Willingness to pay for social health insurance by the self-employed in Port Harcourt, Rivers State; A Contingent Valuation Approach. *Asian J Adv Res Rep*, researchgate.net, cited by 5 (1.25 per year)
67. OA Babatunde, TM Akande, AG Salaudeen, ... (2012) Willingness to pay for community health insurance and its determinants among household heads in rural communities in North-Central Nigeria. *International Review of …*, academia.edu, cited by 67 (6.09 per year)
68. R Oktora (2018) Willingness to Pay for National Health Insurance Among Motorcycle Taxi Driver in Depok City, Indonesia. *KnE Life Sciences*, knepublishing.com, cited by 2 (0.40 per year)
69. G Abdulganiyu, K Muhammad, U Ibrahim, ... (2018) Awareness and Willingness to Pay for Community Based Health Insurance Scheme in North-Western Nigeria. *Bangladesh Journal of …*, banglajol.info, cited by 3 (0.60 per year)
70. NA Nurlia, B Murti, DG Tamtomo (2021) Factors Correlated with Willingness and Compliance to Pay National Health Insurance Premium in Jember Regency. *Journal of Health Policy and …*, thejhpm.com
71. AMM Salameh, MH Juni, KS Hayati (2015) Willingness to pay for social health insurance among academic staff of a public University in Malaysia. *International Journal of Public Health and …*, cited by 12 (1.50 per year)
72. Y Almualm, SE Alkaff, S Aljunid, ... (2013) Factors influencing support for National Health Insurance among patients attending specialist clinics in Malaysia. *Global journal of health …*, ncbi.nlm.nih.gov, cited by 18 (1.80 per year)
73. J Richardson (1999) The role of willingness-to-pay in resource allocation in a National Health Scheme., monash.edu, cited by 7 (0.29 per year)
74. JA Khan (2012) Impact of education on informal workers willingness-to-pay and knowledge of health insurance. *Research Paper*, impactinsurance.org, cited by 8 (0.73 per year)
75. J Ataguba, EH Ichoku, W Fonta (2008) Estimating the willingness to pay for community healthcare insurance in rural Nigeria. *Available at SSRN 1266163*, papers.ssrn.com, cited by 53 (3.53 per year)
76. AV Bustamante, G Ojeda, X Castañeda (2008) Willingness to pay for cross-border health insurance between the United States and Mexico. *Health Affairs*, healthaffairs.org, doi:10.1377/hlthaff.27.1.169, cited by 75 (5.00 per year)
77. A Giwa, M Kabir, II Umar, B Lawal, ... (2018) Awareness and willingness to pay for community based health insurance scheme in North-Western Nigeria. *… of Pharmaceutical & …*, jphs.tms.iau.ir, cited by 2 (0.40 per year)
78. JJ Miti, M Perkio, A Metteri, S Atkins (2021) Factors associated with willingness to pay for health insurance and pension scheme among informal economy workers in low-and middle-income countries: a …. *International Journal of Social …*, emerald.com, doi:10.1108/IJSE-03-2020-0165, cited by 9 (4.50 per year)
79. E Badu, P Agyei‐Baffour, ... (2019) Perceived satisfaction with health services under National Health Insurance Scheme: Clients' perspectives. *… journal of health …*, Wiley Online Library, doi:10.1002/hpm.2711, cited by 15 (3.75 per year)
80. JO Bamidele, WO Adebimpe (2012) Awareness, attitude and willingness of Artisans in Osun State Southwestern Nigeria to participate in community based health insurance. *Journal of Community Medicine and Primary …*, ajol.info, cited by 29 (2.64 per year)
81. AD Intiasari, B Aji, S Masfiah, L Trisnantoro, ... (2019) A study of ability to pay and willingness to pay of national health insurance voluntary participant in rural area. *Ann Trop Public …*, academia.edu, cited by 1 (0.25 per year)
82. D Edoh, A Brenya (2002) A community-based feasibility study of national health insurance scheme in Ghana. *African journal of health sciences*, ajol.info, cited by 11 (0.52 per year)
83. C Tundui, R Macha (2014) Social capital and willingness to pay for community based health insurance: empirical evidence from rural Tanzania. *Journal of Finance and Economics*, academia.edu, cited by 23 (2.56 per year)
84. H Dong, B Kouyate, R Snow, F Mugisha, R Sauerborn (2003) Gender's effect on willingness-to-pay for community-based insurance in Burkina Faso. *Health Policy*, Elsevier, cited by 97 (4.85 per year)
85. AS Oyekale, CG Eluwa (2009) Utilization of health care and health insurance among rural households in Irewole Local Government, Osun state, Nigeria. *International Journal of Tropical …*, makhillpublications.co, cited by 22 (1.57 per year)
86. NS Alharbi (2021) Determinants of Willingness to pay for Employment-Based Health Insurance Among Governmental School Workers in Saudi Arabia. *INQUIRY: The Journal of Health Care …*, journals.sagepub.com, doi:10.1177/00469580211060790, cited by 2 (1.00 per year)
87. P Zweifel (2001) On the use of willingness-to-pay studies in health. *… Zeitschrift für Volkswirtschaft und Statistik= Swiss …*, zora.uzh.ch, cited by 6 (0.27 per year)
88. WY Chen, C Chi, YH Lin (2007) The Willingness to Pay for the Health Care Under Taiwan's National Health Insurance. *iHEA 2007 6th World Congress …*, papers.ssrn.com, cited by 1 (0.06 per year)
89. DA Nugraheni, S Satibi, SA Kristina, ... (2022) Factors Associated with Willingness to Pay for Cost-Sharing under Universal Health Coverage Scheme in Yogyakarta, Indonesia: A Cross-Sectional Survey. *International Journal of …*, mdpi.com
90. P Chiwire, SM Evers, H Mahomed, ... (2021) Willingness to pay for primary health care at public facilities in the Western Cape Province, Cape Town, South Africa. *Journal of Medical …*, Taylor &Francis, doi:10.1080/13696998.2021.1877147, cited by 5 (2.50 per year)
91. I Phiri, W Masanjala, HJ Rösner, ... (2012) Willingness to pay for micro health insurance in Malawi. *Handbook of Micro …*, books.google.com, cited by 5 (0.45 per year)
92. M Negera, D Abdisa (2022) Willingness to pay for community based health insurance scheme and factors associated with it among households in rural community of South West Shoa …. *BMC Health …*, bmchealthservres.biomedcentral …, doi:10.1186/s12913-022-08086-z, cited by 1 (1.00 per year)
93. I Adisah-Atta (2017) Financing health care in Ghana: are Ghanaians willing to pay higher taxes for better health care? Findings from Afrobarometer. *Social sciences*, mdpi.com, cited by 28 (4.67 per year)
94. CH Tang, JT Liu, CW Chang, WY Chang (2007) Willingness to pay for drug abuse treatment: results from a contingent valuation study in Taiwan. *Health Policy*, Elsevier, cited by 41 (2.56 per year)
95. A Behzad, I Sinai, O Sayedi, K Alawi, F Farewar, ... (2022) Willingness and ability to pay for health insurance in Afghanistan. *Health Policy OPEN*, Elsevier
96. A Nanna (2011) Health insurance in developing countries: willingness to pay for health insurance in Thailand using discrete choice experiment methods., espace.curtin.edu.au, cited by 8 (0.67 per year)
97. W Witati, PI Putri (2020) Determinant of willingness to pay health insurance contribution to informal workers. *Economics Development Analysis Journal*, journal.unnes.ac.id, cited by 2 (0.67 per year)
98. IS Saimy, MH Juni, AM Rosliza (2016) Willingness to pay for health insurance and its associated factors among staff of local authorities in Petaling District, Selangor, 2016. *International Journal of Public Health and Clinical …*, cited by 4 (0.57 per year)
99. A Ijeoma, O Adebayo, O Babatunde, ... (2019) Community based health insurance as a viable option for health financing: An assessment of household willingness to pay in Lagos, Nigeria. *Journal of Public …*, academicjournals.org, cited by 5 (1.25 per year)
100. OF Omonira, AS Oyekale+ (2012) Households' Willingness to Pay (WTP) for the National Health Insurance Scheme (NHIS): The Case of Ojo Local Government Area of Lagos State, Nigeria. *Life Science Journal*, Citeseer, cited by 1 (0.09 per year)
101. SEW Puteh, SNA Ahmad, ... (2017) Patients' willingness to pay for their drugs in primary care clinics in an urbanized setting in Malaysia: a guide on drug charges implementation. *Asia Pacific Family …*, apfmj.biomedcentral.com, doi:10.1186/s12930-017-0035-5, cited by 16 (2.67 per year)
102. M Abu‐Zaineh, O Chanel, ... (2022) Estimating willingness to pay for public health insurance while accounting for protest responses: A further step towards universal health coverage in Tunisia?. *The International Journal …*, Wiley Online Library, doi:10.1002/hpm.3505
103. D Acharya, B Devkota, R Adhikari (2018) Willingness to pay for family health insurance: evidence from Baglung and Kailali districts of Nepal. *Global Journal of Health …*, academia.edu, cited by 8 (1.60 per year)
104. AW Kaso, A Haji, HE Hareru, A Hailu (2022) Is Ethiopian community-based health insurance affordable? Willingness to pay analysis among households in South Central, Ethiopia. *Plos one*, journals.plos.org
105. OE Elegbede, KA Durowade, ... (2022) Assessment of willingness to pay for community-based health insurance among artisans in a selected community of Ekiti State, Southwest Nigeria. *Ibom Medical …*, ojs.ibommedicaljournal.org, cited by 2 (2.00 per year)
106. AS Oyekale, A Adeyeye (2012) Rural households' Awareness and willingness to pay for national health insurance scheme (NHIS) in Ilesha West Local Government Area, Osun State Nigeria: A …. *Life Science Journal*, Citeseer, cited by 1 (0.09 per year)
107. M Granberg, M Wikland, L Nilsson, ... (1995) Couples' willingness to pay for IVF/ET. *Acta obstetricia et …*, Wiley Online Library, doi:10.3109/00016349509008938, cited by 52 (1.86 per year)
108. M Thavorncharoensap, ... (2013) Estimating the willingness to pay for a quality-adjusted life year in Thailand: does the context of health gain matter?. *ClinicoEconomics …*, Taylor &Francis, doi:10.2147/CEOR.S38062, cited by 98 (9.80 per year)
109. JO Bock, D Heider, H Matschinger, H Brenner, ... (2016) Willingness to pay for health insurance among the elderly population in Germany. *The European Journal of …*, Springer, doi:10.1007/s10198-014-0663-8, cited by 17 (2.43 per year)
110. A Igarashi, R Goto, ... (2019) Willingness to pay for QALY: perspectives and contexts in Japan. *Journal of Medical …*, Taylor &Francis, doi:10.1080/13696998.2019.1639186, cited by 15 (3.75 per year)
111. A Sana, F Rida, I Tayyaba, M Masooma, ... (2020) Willingness to pay for community-based healthcare insurance schemes in developing countries: a case of Lahore, Pakistan. *Ethiopian Journal of …*, ajol.info, cited by 7 (2.33 per year)
112. EF Adebayo, OA Uthman, ... (2015) A systematic review of factors that affect uptake of community-based health insurance in low-income and middle-income countries. *BMC health …*, bmchealthservres.biomedcentral …, doi:10.1186/s12913-015-1179-3, cited by 181 (22.63 per year)
113. MS Feldstein (1971) A new approach to national health insurance. *The Public Interest*, search.proquest.com, cited by 123 (2.37 per year)
114. CH Lee, H Lim, Y Kim, S Yoon, YS Park, ... (2017) Analysis of new patient's willingness to pay additional costs for securing satisfactory consultation time. *Health policy and …*, koreascience.or.kr, cited by 11 (1.83 per year)
115. DO IBIRONGBE, OE ELEGBEDE, T MICHAEL, ... (2021) Awareness and willingness to pay for community health insurance scheme among rural households in Ekiti State, Nigeria. *Pac J Med …*, researchgate.net, cited by 1 (0.50 per year)
116. F Meng, Z Ji, F Song, T Bai, X Fan, D Wang (2020) Patients' familiarity with, trust in and willingness to pay for traditional Chinese medicine in Chinese community health care centres. *European Journal of …*, Elsevier, cited by 9 (3.00 per year)
117. AB Usman, A Bukola (2013) Willingness to pay for community based health care financing scheme: a comparative study among rural and urban households in Osun State, Nigeria. *J Dent Med Sci*, academia.edu, cited by 39 (3.90 per year)
118. SR Eastaugh (2000) Willingness to pay in treatment of bleeding disorders. *International journal of technology assessment in …*, cambridge.org, cited by 35 (1.52 per year)
119. B Byambajav (2022) Awareness and Willingness to pay for Private health insurance: A study of Mongolian living in South Korea., ir.ymlib.yonsei.ac.kr
120. MK Hyun (2023) Willingness to pay for integrative healthcare services to treat sleep disturbances: Evidence from a nationwide survey. *European Journal of Integrative Medicine*, Elsevier
121. NK Putri, AD Laksono, N Rohmah (2023) Predictors of national health insurance membership among the poor with different education levels in Indonesia. *BMC Public Health*, Springer, doi:10.1186/s12889-023-15292-9
122. EW Anbesu, OA Ebrahim, ... (2022) Willingness to pay for community-based health insurance and associated factors in Ethiopia: A systematic review and meta-analysis. *SAGE Open Medicine*, journals.sagepub.com, doi:10.1177/20503121221135876
123. AT Gessesse, AA Berhe, MG Tilahun, ... (2020) Factors associated with willingness to pay for social health insurance among government employees in Tigrai region, Northern Ethiopia. *…*, eajahme.mzumbe.ac.tz, cited by 3 (1.00 per year)
124. EP Sari (2022) The influence of willing to pay on the compliance with paying the National Health Insurance (NHI) contribution of independent NHI participants during the covid-19 …. *GSC Advanced Research and Reviews*, gsconlinepress.com
125. ED Okoffo, EK Denkyirah, DT Adu, ... (2016) A double-hurdle model estimation of cocoa farmers' willingness to pay for crop insurance in Ghana. *…*, springerplus.springeropen.com, doi:10.1186/s40064-016-2561-2, cited by 62 (8.86 per year)
126. J Oh, Y Ko, A Baer Alley, S Kwon (2015) Participation of the lay public in decision-making for benefit coverage of national health insurance in South Korea. *Health systems &reform*, Taylor &Francis, doi:10.4161/23288604.2014.991218, cited by 37 (4.63 per year)
127. A Sahriana, DS Marzuki, MY Abadi (2019) Analysis of Ability to Pay National Health Insurance Contributions to Communities on Lakkang Island, Makassar City in 2017. *SCOPUS IJPHRD CITATION …*, researchgate.net, cited by 4 (1.00 per year)
128. A Dewi, AGMB Mukti, B Mukti (2014) Differences in attitude of urban and rural residents in accepting national health insurance. *Journal of Biology, Agriculture and …*, academia.edu, cited by 7 (0.78 per year)
129. N Asiza, MR Aidillah, ... (2022) Analysis of Ability to Pay and Willingness to Pay PBPU Participants Who Are In Arrears of National Health Insurance Contributions in Samarinda Ulu District. *Indonesian Journal of …*, jurnal.itkeswhs.ac.id
130. RA Falaki, MH Juni, AM Rosliza (2017) FACTORS AFFECTING WILLINGNESS TO PAY FOR COMMUNITY BASED HEALTH INSURANCE AMONG SECONDARY SCHOOL TEACHERS OF KATSINA …. *International Journal of Public …*, researchgate.net, cited by 1 (0.17 per year)
131. R Ghimire, S Wagle (2021) Willingness to pay and expected benefits for social health insurance: a cross-sectional study at Pokhara metropolitan city. *MedS Alliance Journal of Medicine and Medical …*, nepjol.info, cited by 2 (1.00 per year)
132. A Narawi, M Norhaizam, AS Ting, ... (2018) Willingness to Pay (WTP) For National Social Health Insurance Scheme in Sarawak, Malaysia: A Contingent Valuation Study.. *Global Business & …*, search.ebscohost.com, cited by 1 (0.20 per year)
133. OA Bolarinwa, S Ameh, C Ochimana, ... (2021) Willingness and ability to pay for healthcare insurance: a cross-sectional study of Seven Communities in East and West Africa (SevenCEWA). *PLOS Global Public …*, journals.plos.org, cited by 1 (0.50 per year)
134. N Namuhani (2019) ACCEPTABILITY AND WILLINGNESS TO PAY FOR UGANDA'S PROPOSED NATIONAL HEALTH INSURANCE SCHEME AMONG INFORMAL SECTOR WORKERS …., dspace.mak.ac.ug
135. AD Laksono, ZK Nantabah, ... (2022) Barriers to expanding the national health insurance membership in Indonesia: who should the target?. *Journal of primary …*, journals.sagepub.com, doi:10.1177/21501319221111112, cited by 5 (5.00 per year)
136. AS Serge Oga (2019) Willingness to pay for national health insurance in Ivory Coast. *PharmacoEconomics &Outcomes News*, Springer, doi:10.1007/s40274-019-5760-6
137. CW Muheki (1998) Willingness to pay for social health insurance: a case study of Kampala (Uganda)., open.uct.ac.za, cited by 4 (0.16 per year)
138. K Ohshige, S Mizushima, O Tochikubo (2004) Willingness to pay for a public health checkup program: assessment by the travel cost method. *Nihon Koshu Eisei Zasshi …*, jstage.jst.go.jp, cited by 4 (0.21 per year)
139. H Ko, H Kim, C Yoon, C Kim (2018) Social capital as a key determinant of willingness to join community-based health insurance: a household survey in Nepal. *Public Health*, Elsevier, cited by 25 (5.00 per year)
140. D Gyrd-Hansen (2005) Willingness to pay for a QALY: theoretical and methodological issues. *Pharmacoeconomics*, Springer, doi:10.2165/00019053-200523050-00002, cited by 137 (7.61 per year)
141. YT Chen, YH Ying, K Chang, YH Hsieh (2016) Study of patients' willingness to pay for a cure of chronic obstructive pulmonary disease in Taiwan. *International journal of …*, mdpi.com, cited by 12 (1.71 per year)
142. DY OH, B Crawford, SB KIM, ... (2012) Evaluation of the willingness‐to‐pay for cancer treatment in Korean metastatic breast cancer patients: A multicenter, cross‐sectional study. *Asia‐Pacific Journal …*, Wiley Online Library, doi:10.1111/j.1743-7563.2012.01546.x, cited by 15 (1.36 per year)
143. L Gwokorok (2021) Health Believes and Willingness to Pay (WTP) for Social Health Insurance in Post Conflicts Fragile State South Sudan., researchsquare.com, cited by 2 (1.00 per year)
144. XH Ying, TW Hu, J Ren, W Chen, K Xu, ... (2007) Demand for private health insurance in Chinese urban areas. *Health …*, Wiley Online Library, doi:10.1002/hec.1206, cited by 60 (3.75 per year)
145. M Wahidin, TJ Angkasawati (2021) Ability to pay and willingness to pay for insurance from tobacco expenditure among non-members of National Health Insurance in Indonesia, 2019. *Tobacco Induced …*, tobaccoinduceddiseases.org
146. E Witcahyo (2018) Relationship Between Total Incomes with Willingness to Pay in National Health Insurance on Coffee Farmer in Jember., scitepress.org
147. T Tawiah, K Malam, A Kwarteng, C Bart-Plange, ... (2018) Improving the first-line treatment of febrile illnesses in Ghana: willingness to pay for malaria rapid diagnostic tests at licensed chemical shops in the Kintampo …. *Cost Effectiveness and …*, Springer, doi:10.1186/s12962-018-0090-2, cited by 9 (1.80 per year)
148. CRC Kouakou, TG Poder (2022) Willingness to pay for a quality-adjusted life year: a systematic review with meta-regression. *The European Journal of Health Economics*, Springer, doi:10.1007/s10198-021-01364-3, cited by 13 (13.00 per year)
149. S Kruiroongroj, ... (2014) Knowledge, acceptance, and willingness to pay for human papilloma virus (HPV) vaccination among female parents in Thailand. *Asian Pacific Journal of …*, koreascience.or.kr, cited by 39 (4.33 per year)
150. R Puurbalanta, M Adjei, V Afosaa (2020) Ghana's National Health Insurance Scheme: An Ordinal Probit Valuation of Willingness to Pay Higher Premiums for Improved Services. *American Journal of …*, researchgate.net
151. MG Garedew, SO Sinkie, DM Handalo, ... (2020) Willingness to join and pay for community-based health insurance among rural households of selected districts of Jimma zone, southwest Ethiopia. *ClinicoEconomics …*, Taylor &Francis, doi:10.2147/CEOR.S227934, cited by 17 (5.67 per year)
152. J Jeetoo, VC Jaunky (2022) Willingness to pay to improve quality of public healthcare services in Mauritius. *Healthcare*, mdpi.com, cited by 6 (6.00 per year)
153. RK Alhassan, ... (2022) Financing COVID-19 vaccination in sub-Saharan Africa: lessons from a nation-wide willingness to pay (WTP) survey in Ghana. *BMC Public …*, bmcpublichealth.biomedcentral.com, doi:10.1186/s12889-022-13602-1, cited by 4 (4.00 per year)
154. TA Agago, M Woldie, S Ololo (2014) Willingness to join and pay for the newly proposed social health insurance among teachers in Wolaita Sodo town, South Ethiopia. *Ethiopian journal of health sciences*, ajol.info, cited by 58 (6.44 per year)
155. F Mekonnen Degie, Y Agumas Ambelie, ... (2021) Willingness to pay for social health insurance and its predictors among government employees in Mujja Town, Ethiopia. *The Scientific World …*, hindawi.com, cited by 3 (1.50 per year)
156. A Hajek, C Enzenbach, K Stengler, ... (2020) Determinants of Willingness to Pay for Health Insurance in Germany—Results of the Population-Based Health Study of the Leipzig Research Centre for …. *Frontiers in Public …*, frontiersin.org, doi:10.3389/fpubh.2020.00456, cited by 5 (1.67 per year)
157. HJ Choi, EW Lee (2019) Methodology of estimating socioeconomic burden of disease using National health insurance (NHI) data. *Eval Heal Serv*, books.google.com, cited by 6 (1.50 per year)
158. A Victoor, J Hansen, ME van den Akker-van, ... (2014) Choosing your health insurance package: A method for measuring the public's preferences for changes in the national health insurance plan. *Health Policy*, Elsevier, cited by 6 (0.67 per year)
159. E Kalyango (2021) Household preferences and willingness to pay for health insurance in Kawempe division: a discrete choice experiment.., 196.43.133.114
160. JM Boaheng, E Amporfu, ... (2019) Determinants of paying national health insurance premium with mobile phone in Ghana: a cross-sectional prospective study. *… for Equity in …*, equityhealthj.biomedcentral.com, doi:10.1186/s12939-019-0946-x, cited by 15 (3.75 per year)
161. Q Zhang, D Ren, X Chang, C Sun, R Liu, ... (2022) Willingness to pay for packaging cancer screening of Chinese rural residents. *Cancer …*, Wiley Online Library, doi:10.1002/cam4.5162
162. H Dong, B Kouyate, J Cairns, R Sauerborn (2003) A comparison of the reliability of the take-it-or-leave-it and the bidding game approaches to estimating willingness-to-pay in a rural population in West Africa. *Social Science &Medicine*, Elsevier, cited by 68 (3.40 per year)
163. IAO Odeyemi (2014) Community-based health insurance programmes and the national health insurance scheme of Nigeria: challenges to uptake and integration. *International journal for equity in health*, Springer, doi:10.1186/1475-9276-13-20, cited by 154 (17.11 per year)
164. MF Alharbi (2022) Willingness to Pay for Employment-Based Health Insurance: A Study among Government Employees in Qassim Region, Saudi Arabia., academia.edu
165. CM Ko, CK Koh, S Kwon (2019) Willingness to pay for family education and counselling services provided by critical care advanced practice nurses. *International Journal of Nursing …*, Wiley Online Library, doi:10.1111/ijn.12782, cited by 1 (0.25 per year)
166. S Aung Awareness and Acceptance on Health Insurance, and Willingness to Pay: A Community Based Study in Myanmar. *researchgate.net*
167. A Hidayat (2022) … KAKI LIMA DI PANTAI LOSARI= DETERMINANTS OF ABILITY AND WILLINGNESS TO PAY NATIONAL HEALTH INSURANCE CONTRIBUTIONS TO STREET …., repository.unhas.ac.id
168. FE Astrilia, YH Hadiwiardjo, G Soeryo (2021) Factors Affecting Willingness to Pay for Outpatient Services at RSU Tangerang Selatan. *Disease Prevention and Public …*, core.ac.uk
169. SO OJEZELE (2015) WILLINGNESS TO PAY FOR VOLUNTARY CONTRIBUTOR SOCIAL HEALTH INSURANCE AMONG RURAL DWELLERS IN ERUWA, OYO STATE, NIGERIA., library.adhl.africa
170. CY Myint, M Pavlova, W Groot (2019) Health insurance in Myanmar: Knowledge, perceptions, and preferences of Social Security Scheme members and general adult population. *The international journal of …*, Wiley Online Library, doi:10.1002/hpm.2643, cited by 12 (3.00 per year)
171. H Yasunaga, H Ide, T Imamura, ... (2006) Willingness to pay for health care services in common cold, retinal detachment, and myocardiac infarction: an internet survey in Japan. *BMC health …*, bmchealthservres.biomedcentral …, doi:10.1186/1472-6963-6-12, cited by 48 (2.82 per year)
172. K Mulenga, F Booysen (2022) Inequalities in willingness to pay for Zambia's National Social Health Insurance Scheme., researchsquare.com
173. DM Djahini-Afawoubo, EH Atake (2018) Extension of mandatory health insurance to informal sector workers in Togo. *Health economics review*, Springer, doi:10.1186/s13561-018-0208-4, cited by 13 (2.60 per year)
174. DC Arhin (1998) Willingness to pay for rural health insurance: evidence from three African countries., search.proquest.com, cited by 3 (0.12 per year)
175. MK Al-Hanawi, O Alsharqi, K Vaidya (2020) Willingness to pay for improved public health care services in Saudi Arabia: a contingent valuation study among heads of Saudi households. *Health Economics, Policy and …*, cambridge.org, cited by 24 (8.00 per year)
176. N Havet, M Morelle, R Remonnay, ... (2012) Cancer patients' willingness to pay for blood transfusions at home: results from a contingent valuation study in a French cancer network. *The European Journal of …*, Springer, doi:10.1007/s10198-011-0328-9, cited by 32 (2.91 per year)
177. A Baillon, A Kraft, O O'donnell, ... (2022) A behavioral decomposition of willingness to pay for health insurance. *Journal of Risk and …*, Springer, doi:10.1007/s11166-022-09371-2, cited by 3 (3.00 per year)
178. CE Mbada, SO Mamud, AC Odole, ... (2019) Development and clinimetric testing of willingness to pay tool for physiotherapy. *Physical Therapy …*, Taylor &Francis, doi:10.1080/10833196.2019.1627703, cited by 4 (1.00 per year)
179. RK Basaza, JH Kiconco, EP Kyasiimire, ... (2022) Determinants of Willingness to Pay for Community Health Insurance Among Commercial Motorcyclists in Kampala City, Uganda: A Contingency Valuation Study. *Journal of Research and …*, jrh.gmu.ac.ir
180. V Birungi (2014) Willingness to Pay for Community Health Insurance Among Households in Wakiso District, Uganda.., dspace.ciu.ac.ug, cited by 1 (0.11 per year)
181. N Gulati, B Chaudhuri (2020) Role of information in determining the willingness to pay for health insurance. *Contemporary Issues in Sustainable …*, taylorfrancis.com, doi:10.4324/9781003141020-20
182. K Darmawan, SA Kristina (2020) PIH14 Perception of Seriousness, Knowledge, and Willingness to Pay for Cervical Cancer Screening Among Women in Yogyakarta Province, Indonesia. *Value in Health …*, valuehealthregionalissues.com, cited by 1 (0.33 per year)
183. V Boima, K Agyabeng, V Ganu, D Dey, E Yorke, ... (2020) Willingness to pay for kidney transplantation among chronic kidney disease patients in Ghana. *PloS one*, journals.plos.org, cited by 3 (1.00 per year)
184. T Shamsi (2021) Burden of breast cancer in Bangladesh‐current and future and financing treatment with link to willingness to pay. *Int J Community Med Public Heal*, researchgate.net, cited by 1 (0.50 per year)
185. D Mpuuga (2019) Determinants of demand for health insurance in Uganda., Makerere University
186. VR Fuchs (1991) National health insurance revisited. *Health Affairs*, healthaffairs.org, doi:10.1377/hlthaff.10.4.7, cited by 59 (1.84 per year)
187. I Nozaki, K Wada, ... (2017) Public views of health insurance in Japan during the era of attaining universal health coverage: a secondary analysis of an opinion poll on health insurance in …. *Journal of Public Health …*, journals.sagepub.com, doi:10.4081/jphr.2017.884, cited by 2 (0.33 per year)
188. W Hsu, CH Yang, WP Fan (2021) A study of patients' willingness to pay for a basic outpatient copayment and medical service quality in Taiwan. *… of Environmental Research and Public Health*, mdpi.com, cited by 3 (1.50 per year)
189. CO Kim, S Jang (2020) Who are the people willing to pay for physician home visits?. *Journal of Korean Medical Science*, synapse.koreamed.org, cited by 8 (2.67 per year)
190. HB Singu (2021) Willingness to pay for customized health insurance package: An exploratory study in a developing country. *SMART Journal of Business Management Studies*, indianjournals.com
191. SF Tarigan, ML Dondo (2021) Behavior Paying Premium to the Independent Participants in Healthcare Social Insurance Administration Office. *KEMAS: Jurnal Kesehatan Masyarakat*, journal.unnes.ac.id, cited by 1 (0.50 per year)
192. T Dartanto, JF Rezki, CH Siregar, H Bintara, ... (2015) Expanding universal health coverage in the presence of informality in Indonesia: challenges and policy implications., ideas.repec.org, cited by 5 (0.63 per year)
193. JT Liu, MW Tsou, JK Hammitt (2009) Willingness to pay for weight-control treatment. *Health Policy*, Elsevier, cited by 23 (1.64 per year)
194. G Daniyarova (2020) Factors influencing willingness to pay for compulsory social health insurance among informal sector workers in Kazakhstan., ir.ymlib.yonsei.ac.kr
195. G Iskrov, D Greenberg, I Yakimov, H Cholakova, ... (2019) What is the value of innovative pharmaceutical therapies in oncology and hematology? A willingness-to-pay study in Bulgaria. *Value in Health …*, Elsevier, cited by 7 (1.75 per year)
196. OA Uche, LO Akanni, AO Ruth (2016) WILLINGNESS TO PAY FOR COMMUNITY BASED HEALTH INSURANCE SCHEME AMONG PREGNANT WOMEN IN LAGOS STATE.. *African Journal of Health Economics*, ajhe.org.in
197. JN Orem, CM Zikusooka (2010) Health financing reform in Uganda: How equitable is the proposed National Health Insurance scheme?. *… for equity in …*, equityhealthj.biomedcentral.com, doi:10.1186/1475-9276-9-23, cited by 66 (5.08 per year)
198. HC Lang, K Chang, YH Ying (2012) QUALITY OF LIFE, TREATMENTS, AND PATIENTS'WILLINGNESS TO PAY FOR A COMPLETE REMISSION OF CERVICAL CANCER IN TAIWAN. *Health economics*, Wiley Online Library, doi:10.1002/hec.1786, cited by 24 (2.18 per year)
199. MH Huang, CF Tsai, WJ Lee, WF Wang, ... (2021) Caregivers' willingness to pay for Alzheimer's disease medications in Taiwan. *Journal of the …*, journals.lww.com, cited by 2 (1.00 per year)
200. MJ Lee, EK Yoo, BJ Seo (2014) A study on willingness to pay of diabetic patients for nursing intervention using telecommunication. *International Journal of Bio-Science and Bio …*, gvpress.com, cited by 4 (0.44 per year)
201. **Scopus (n = 1****9) – using “perish or publish” software:**

**Willingness to pay "national health insurance" [title]**

*Publish or Perish 8.8.4275.8412 (basic report)
WinPosix (x64) edition, running on WinPosix 10.0.19045 (x64)*

**Search terms**

**Title words:** Willingness to pay "national health insurance"
**Years:** all

**Data retrieval**

**Data source:** Scopus
**Search date:** 2023-03-31 15:39:48 +00300
**Cache date:** 2023-03-31 12:39:49 +00300
**Search result:** [0] No error

***Important:*** *This data source returns only one author per article; this affects the calculation of per-author metrics.*

**Metrics**

**Reference date:** 2023-03-31 12:39:49 +00300
**Publication years:** 2008-2023
**Citation years:** 15 (2008-2023)
**Papers:** 19
**Citations:** 119
**Citations/year:** 7.93 (acc1=7, acc2=4, acc5=1, acc10=0, acc20=0)
**Citations/paper:** 6.26
**Authors/paper:** 1.00/1.0/1 (mean/median/mode)
**Age-weighted citation rate:** 18.29 (sqrt=4.28), 18.29/author
**Hirsch h-index:** 6 (a=3.31, m=0.40, 108 cites=90.8% coverage)
**Egghe g-index:** 10 (g/h=1.67, 117 cites=98.3% coverage)
**PoP hI,norm:** 6
**PoP hI,annual:** 0.40
**Fassin hA-index:** 3

**Results**

1. M.K. Al-Hanawi (2018) Investigating the Willingness to Pay for a Contributory National Health Insurance Scheme in Saudi Arabia: A Cross-sectional Stated Preference Approach. *Applied Health Economics and Health Policy* 16(2), pp. 259-271, ISSN 1175-5652, doi:10.1007/s40258-017-0366-2, cited by 26 (5.20 per year)
2. R. Adams (2015) Willingness to participate and Pay for a proposed national health insurance in St. Vincent and the grenadines: A cross-sectional contingent valuation approach. *BMC Health Services Research* 15(1), ISSN 1472-6963, doi:10.1186/s12913-015-0806-3, cited by 22 (2.75 per year)
3. R. Basaza (2017) Willingness to pay for National Health Insurance Fund among public servants in Juba City, South Sudan: A contingent evaluation. *International Journal for Equity in Health* 16(1), ISSN 1475-9276, doi:10.1186/s12939-017-0650-7, cited by 18 (3.00 per year)
4. M.A. González Block (2014) Redressing the limitations of the affordable care act for Mexican immigrants through bi-national health insurance: A willingness to pay study in Los Angeles. *Journal of Immigrant and Minority Health* 16(2), pp. 179-188, ISSN 1557-1912, doi:10.1007/s10903-012-9712-5, cited by 18 (2.00 per year)
5. Oyekale (2012) Factors influencing households' willingness to pay for National Health Insurance Scheme (NHIS) in Osun State, Nigeria. *Studies on Ethno-Medicine* 6(3), pp. 167-172, ISSN 0973-5070, doi:10.1080/09735070.2012.11886435, cited by 13 (1.18 per year)
6. H. Lang (2008) Willingness to pay to sustain and expand National Health Insurance services in Taiwan. *BMC Health Services Research* 8, ISSN 1472-6963, doi:10.1186/1472-6963-8-261, cited by 11 (0.73 per year)
7. W. Chen (2011) The willingness to pay for the health care under Taiwan's national health insurance system. *Applied Economics* 43(9), pp. 1113-1123, ISSN 0003-6846, doi:10.1080/00036840802600228, cited by 4 (0.33 per year)
8. A.D. Intiasari (2019) A study of ability to pay and willingness to pay of national health insurance voluntary participant in rural area. *Annals of Tropical Medicine and Public Health* 22(11), ISSN 1755-6783, doi:10.36295/ASRO.2019.221124, cited by 3 (0.75 per year)
9. P. Agyei-Baffour (2022) Socio-Demographic Predictors of Willingness to Pay for Premium of National Health Insurance: A Cross-sectional Survey of Six Districts in Sierra Leone. *International Journal of Health Policy and Management* 11(8), pp. 1451-1458, ISSN 2322-5939, doi:10.34172/ijhpm.2021.50, cited by 1 (1.00 per year)
10. R.T.H. Tan (2022) Willingness to Pay for National Health Insurance: A Contingent Valuation Study Among Patients Visiting Public Hospitals in Melaka, Malaysia. *Applied Health Economics and Health Policy* 20(2), pp. 255-267, ISSN 1175-5652, doi:10.1007/s40258-021-00691-z, cited by 1 (1.00 per year)
11. A.S.S. Oga (2019) Diabetic and cardiovascular patients' willingness to pay for upcoming national health insurance scheme in Côte d'Ivoire. *Health Economics Review* 9(1), ISSN 2191-1991, doi:10.1186/s13561-019-0225-y, cited by 1 (0.25 per year)
12. O. Omonira (2012) Households' Willingness to Pay (WTP) for the National Health Insurance Scheme (NHIS): The case of Ojo local government area of Lagos State, Nigeria. *Life Science Journal* 9(4), pp. 3873-3877, ISSN 1097-8135, cited by 1 (0.09 per year)
13. H. Njie (2023) Willingness to pay for a National Health Insurance Scheme in The Gambia: a contingent valuation study. *Health policy and planning* 38(1), pp. 61-73, ISSN 1460-2237, doi:10.1093/heapol/czac089
14. R. Mansur (2022) Using Tree-Based Algorithm to Predict Informal Workers' Willingness to Pay National Health Insurance after Tele-Collection. *2022 10th International Conference on Information and Communication Technology, ICoICT 2022*, pp. 23-28, doi:10.1109/ICoICT55009.2022.9914901
15. H. Hasan (2022) Willingness to Pay for the National Health Insurance Scheme: A Cross-sectional study in Sarawak, Malaysia. *Bangladesh Journal of Medical Science* 21(3), pp. 577-589, ISSN 2223-4721, doi:10.3329/bjms.v21i3.59571
16. Alharbi (2022) Willingness to pay for a National Health Insurance (NHI) in Saudi Arabia: a cross-sectional study. *BMC Public Health* 22(1), ISSN 1471-2458, doi:10.1186/s12889-022-13353-z
17. K.T. Han (2021) Is Korean society prepared for the financial burden of novel anticancer drugs? A survey of willingness to pay among National Health Insurance beneficiaries. *Supportive Care in Cancer* 29(11), pp. 6681-6688, ISSN 0941-4355, doi:10.1007/s00520-021-06091-2
18. R. Adams (2016) Erratum to: Willingness to participate and Pay for a proposed national health insurance in St. Vincent and the grenadines: A cross-sectional contingent valuation approach (BMC Health Services Research (2016) 16 (73)). *BMC Health Services Research* 16(1), ISSN 1472-6963, doi:10.1186/s12913-016-1322-9
19. Samuel Oyekale (2012) Rural households' awareness and willingness to pay for national health insurance scheme (NHIS) in ilesha west local government area, osun State Nigeria: A recursive bivariate probit approach. *Life Science Journal* 9(4), pp. 2086-2093, ISSN 1097-8135
20. **Research4Life (R4L) - Hinari (N= 50):**

**Search:** (Willingness to pay) AND (Social health insurance) AND (Ethiopia)

**Date:** 2023-04-03 (03 April 2023)

**Selected by:**

- **Accessibility:** Full text online, open access, scholarly and per-reviewed
- **Content Type:** Journal article
- **Publication date:** 10 years
- **Discipline:** Public health
- **Subject terms:** Willingness to pay, national health insurance
- **Language:** English

**Results**

1. Hsu, W., Chih-Hao, Y. & Wen-Ping, F. A Study of Patients’ Willingness to Pay for a Basic Outpatient Copayment and Medical Service Quality in Taiwan. *International journal of environmental research and public health* 18, 6604 (2021).
2. Adebayo, E. F., Uthman, O. A., Wiysonge, C. S. & Stern, E. A. A systematic review of factors that affect uptake of community-based health insurance in low-income and middle-income countries. *BMC health services research* 15, 543 (2015).
3. Nguyen, T. T. T., Kasemsup, V., Tiraphat, S. & Srithamrongsawat, S. Acceptability and willingness to pay for influenza vaccination among healthcare professionals in Vietnam. *Asia Pacific journal of health management* 16, 205–214 (2021).
4. Tewele, A., Yitayal, M. & Kebede, A. Acceptance for Social Health Insurance among Health Professionals in Government Hospitals, Mekelle City, North Ethiopia. *Advances in Public Health* 2020, (2020).
5. Zemene, A., Kebede, A., Atnafu, A. & Gebremedhin, T. Acceptance of the proposed social health insurance among government-owned company employees in Northwest Ethiopia: implications for starting social health insurance implementation. *Archives of public health = Archives belges de santé publique* 78, (2020).
6. Hassan, A. Y. I., Cucculelli, M. & Lamura, G. Caregivers’ willingness to pay for digital support services: Comparative survey. *Health policy (Amsterdam)* 130, 104751 (2023).
7. Victoor, A., Hansen, J., van den Akker-Van Marle, M. E. & van den Berg, B. Choosing your health insurance package: A method for measuring the public’s preferences for changes in the national health insurance plan. *Health policy (Amsterdam)* 117, 257–265 (2014).
8. Odeyemi, I. A. O. Community-based health insurance programmes and the National Health Insurance Scheme of Nigeria: challenges to uptake and integration. *International journal for equity in health* 13, 20 (2014).
9. García, L. Y. L. Y. & Cerda, A. A. A. A. Contingent assessment of the COVID-19 vaccine. *Vaccine* 38, 5424–5429 (2020).
10. Debie, A. A., Khatri, R. B. R. B. & Assefa, Y. Y. Contributions and challenges of healthcare financing towards universal health coverage in Ethiopia: a narrative evidence synthesis. *BMC health services research* 22, (2022).
11. Wang, Q., Zhou, Y., Ding, X. & Ying, X. Demand for Long-Term Care Insurance in China. *International journal of environmental research and public health* 15, 6 (2017).
12. Oga, A. S. S., Attia-konan, A. R., Vehi, F. & Kouame, J. Diabetic and cardiovascular patients’ willingness to pay for upcoming national health insurance scheme in Côte d’Ivoire. *Health economics review* 9, (2019).
13. Savitha, B. & Banerjee, S. Education and Experience as Determinants of Micro Health Insurance Enrolment. *International journal of health policy and management* 10, 192 (2021).
14. Obse, A., Ryan, M., Heidenreich, S. & Normand, C. Eliciting preferences for social health insurance in Ethiopia. *Health policy and planning* 31, 1423–1432 (2016).
15. Abu-Zaineh, M., Chanel, O. & Makhloufi, K. Estimating willingness to pay for public health insurance while accounting for protest responses: A further step towards universal health coverage in Tunisia? *The International journal of health planning and management* 37, 2809–2821 (2022).
16. Habib, S. S. & Zaidi, S. Exploring willingness to pay for health insurance and preferences for a benefits package from the perspective of women from low-income households of Karachi, Pakistan. *BMC health services research* 21, 1 (2021).
17. Fite, M. B., Beha, G. A., Gurmessa, T. T. & Roba, K. T. Factors associated with enrollment for community-based health insurance scheme in Western Ethiopia: Case-control study. *PloS one* 16, e0252303 (2021).
18. Nugraheni, D. A., Satibi, S., Kristina, S. A. & Puspandari, D. A. Factors Associated with Willingness to Pay for Cost-Sharing under Universal Health Coverage Scheme in Yogyakarta, Indonesia: A Cross-Sectional Survey. *International journal of environmental research and public health* 19, (2022).
19. Conde, K. K., Camara, A. M., Jallal, M. & Khalis, M. Factors determining membership in community-based health insurance in West Africa: a scoping review. *Global health research and policy* 7, (2022).
20. Yang, X., Zhang, A. & Zhang, F. Farmers’ Heterogeneous Willingness to Pay for Farmland Non-Market Goods and Services on the Basis of a Mixed Logit Model—A Case Study of Wuhan, China. *International journal of environmental research and public health* 16, 3876 (2019).
21. Wanapirak, C., Buddhawongsa, P., Himakalasa, W. & Sarnwong, A. Fetal Down syndrome screening models for developing countries; Part II: Cost-benefit analysis. *BMC health services research* 19, (2019).
22. Alhassan, R. K. R. K., Nketiah-Amponsah, E. E., Immurana, M. M. & Abuosi, A. A. A. A. Financing COVID-19 vaccination in sub-Saharan Africa: lessons from a nation-wide willingness to pay (WTP) survey in Ghana. *BMC public health* 22, (2022).
23. Kalyango, E., Kananura, R. M. & Kiracho, E. E. Household preferences and willingness to pay for health insurance in Kampala City: a discrete choice experiment. *Cost effectiveness and resource allocation* 19, (2021).
24. Zirui Song MD, P. & Sanjay Basu MD, P. Improving Affordability and Equity in Medicare Advantage. *Inquiry (Chicago)* 56, (2019).
25. Tawiah, T., Malam, K., Kwarteng, A. & Bart-Plange, C. Improving the first-line treatment of febrile illnesses in Ghana: willingness to pay for malaria rapid diagnostic tests at licensed chemical shops in the Kintampo area. *Cost effectiveness and resource allocation* 16, (2018).
26. Jarlenski, M. P., Gudzune, K. A., Bennett, W. L. & Cooper, L. A. Insurance Coverage for Weight Loss: Overweight Adults’ Views. *American journal of preventive medicine* 44, 453–458 (2013).
27. Chen Tseng, Y., Jen Wang, I. & Pu, C. Parents’ perception and willingness to maintain provider care continuity for their children under universal health coverage. *AIMS public health* 6, 121–134 (2019).
28. Vera Cruz, G. & Dlamini, P. S. People’s willingness and determinants to use selected tele-consultation public health services in Mozambique. *BMC public health* 21, 947 (2021).
29. Pu, C., Yu-Chen, T., Tang, G.-J. & Yen-Hsiung, L. Perception and Willingness to Maintain Continuity of Care by Parents of Children with Asthma in Taiwan. *International journal of environmental research and public health* 18, 3600 (2021).
30. Putri, N. K., Laksono, A. D. & Rohmah, N. Predictors of national health insurance membership among the poor with different education levels in Indonesia. *BMC public health* 23, (2023).
31. Gerry, C. J., Kaneva, M. & Zasimova, L. Reforming voluntary drug insurance in Russian healthcare: does social solidarity matter? *Health policy (Amsterdam)* 121, 1177 (2017).
32. Kanchebe Derbile, E. & van der Geest, S. Repackaging exemptions under National Health Insurance in Ghana: how can access to care for the poor be improved? *Health policy and planning* 28, 586–595 (2013).
33. Ko, H., Kim, H., Yoon, C. & Kim, C. Social capital as a key determinant of willingness to join community-based health insurance: a household survey in Nepal. *Public health (London)* 160, 52–61 (2018).
34. Jimmy, A. I., Twum, P., Bangura, A. & Larbie, D. Socio-Demographic Predictors of Willingness to Pay for Premium of National Health Insurance: A Cross-sectional Survey of Six Districts in Sierra Leone. *International journal of health policy and management* 11, 1451 (2022).
35. Wang, Y. & Zhang, L. Status of public-private partnership recognition and willingness to pay for private health care in China. *The International journal of health planning and management* 34, e1188–e1199 (2019).
36. Chen, Y.-T., Ying, Y.-H., Chang, K. & Hsieh, Y.-H. Study of Patients’ Willingness to Pay for a Cure of Chronic Obstructive Pulmonary Disease in Taiwan. *International journal of environmental research and public health* 13, (2016).
37. Behzad, A., Sinai, I., Sayedi, O. & Alawi, K. Willingness and ability to pay for health insurance in Afghanistan. *Health Policy OPEN* 3, 100076 (2022).
38. Deksisa, A., Abdo, M., Mohamed, E. & Tolesa, D. Willingness of community based health insurance uptake and associated factors among urban residents of Oromia regional state, Oromia, Ethiopia, a cross-sectional study. *BMC health services research* 20, 1–12 (2020).
39. Sackey, F. G. & Amponsah, P. N. Willingness to accept capitation payment system under the Ghana National Health Insurance Policy: do income levels matter? *Health economics review* 7, 1–13 (2017).
40. Garedew, M. G., Sinkie, S. O., Handalo, D. M. & Salgedo, W. B. Willingness to Join and Pay for Community-Based Health Insurance Among Rural Households of Selected Districts of Jimma Zone, Southwest Ethiopia. *ClinicoEconomics and outcomes research* 12, 45 (2020).
41. Adams, R., Chou, Y.-J. & Pu, C. Willingness to participate and Pay for a proposed national health insurance in St. Vincent and the grenadines: a cross-sectional contingent valuation approach. *BMC health services research* 15, (2015).
42. Wang, J., Lyu, Y., Zhang, H. & Jing, R. Willingness to pay and financing preferences for COVID-19 vaccination in China. *Vaccine* 39, 1968 (2021).
43. Alharbi, A. Willingness to pay for a National Health Insurance (NHI) in Saudi Arabia: a cross-sectional study. *BMC public health* 22, 1 (2022).
44. NOSRATNEJAD, S., RASHIDIAN, A., SARI, A. A. & MORADI, N. Willingness to Pay for Complementary Health Care Insurance in Iran. *Iranian journal of public health* 46, (2017).
45. Basaza, R., Alier, P. K., Kirabira, P. & Ogubi, D. Willingness to pay for National Health Insurance Fund among public servants in Juba City, South Sudan: a contingent evaluation. *International journal for equity in health* 16, 1–10 (2017).
46. Chiwire, P., Evers, S. M., Mahomed, H. & Hiligsmann, M. Willingness to pay for primary health care at public facilities in the Western Cape Province, Cape Town, South Africa. *Journal of medical economics* 24, 162–172 (2021).
47. Batbold, O. & Pu, C. Willingness to pay for private health insurance among workers with mandatory social health insurance in Mongolia. *International journal for equity in health* 20, 1–14 (2021).
48. Nembrini, S., Ceretti, E., Gelatti, U. & Castaldi, S. Willingness to pay for risky lifestyles: results from the Pay for Others (PAY4O) study, Italy. *Public health (London)* 182, 179–184 (2020).
49. Gidey, M. T., Gebretekle, G. B., Hogan, M.-E. & Fenta, T. G. Willingness to pay for social health insurance and its determinants among public servants in Mekelle City, Northern Ethiopia: a mixed methods study. *Cost effectiveness and resource allocation* 17, 2 (2019).
50. Bayked, E. M., Toleha, H. N., Chekole, B. B. & Workneh, B. D. Willingness to pay for social health insurance in Ethiopia: A systematic review and meta-analysis. *Frontiers in public health* 11, (2023).
